# Dynamic positioning of Rpc34 winged helix in RNA polymerase III elongation complex for its stability with implications for reinitiation

**DOI:** 10.1073/pnas.2601775123

**Published:** 2026-06-29

**Authors:** Jheng-Syong Wu, Yu-Chun Lin, Yi-Yu Wei, Hsin-Hung Lin, Yang-Chih Liu, Jen-Wei Chang, I-Ping Tu, Hung-Ta Chen, Wei-Hau Chang

**Affiliations:** ^a^ Institute of Chemistry, Academia Sinica, Taipei 115, Taiwan; ^b^ Institute of Molecular Biology, Academia Sinica, Taipei 115, Taiwan; ^c^ Institute of Statistical Science, Academia Sinica, Taipei 115, Taiwan; ^d^Genomic Research Center, Academia Sinica, Taipei 115, Taiwan; ^e^ Institute of Physics, Academia Sinica, Taipei 115, Taiwan

**Keywords:** RNA polymerase, transcription elongation, single-molecule FRET, protein dynamics, bio-orthogonal chemistry

## Abstract

RNA polymerase III (Pol III) is specialized for the high-throughput synthesis of short RNAs, a capability linked to its unique TFIIE- and TFIIF-like subcomplexes that are stably associated through different stages of transcription. To date, the role of a winged helix domain (WH2) of Rpc34 subunit in the TFIIE-like subcomplex during elongation has remained a conundrum because its density is consistently absent in cryo-EM structures of Pol III elongation complexes (ECs), suggesting its high conformational mobility. In this study, we employed single-molecule Förster resonance energy transfer (smFRET) and nano-positioning triangulation to characterize the dynamics and determine the position of the Rpc34-WH2 domain within transcription-competent but nontranslocating Pol III ECs. To achieve the required site-specific labeling, we developed a chemical biology framework that utilizes azido-carrying unnatural amino acid incorporation and a thiol-capping strategy to eliminate off-target alkyne-thiol cross-reactivity. With the acceptor at Rpc34-WH2 and the donor at a defined position on the DNA template as the reference point, our smFRET results reveal that Rpc34-WH2 dynamically transitions among three discrete states, corresponding to preferred positional sites in downstream, middle, and upstream regions across the DNA-binding cleft. One of these sites coincides with Rpc34-WH2’s position in the preinitiation complex, indicating positional similarity across transcriptional states. Together with prior Pol I and Pol II studies, these findings establish Rpc34-WH2 as a mobile regulatory element that engages the Pol III EC through transient, weak interactions. Additionally, the bio-orthogonal labeling strategy presented here provides a robust, generalizable route for smFRET studies of large, multisubunit protein assemblies.

## Significance

RNA Polymerase III (Pol III) is essential for rapid production of structural RNAs, yet how its mobile components coordinate this high-speed machinery remains unclear. Using a chemical biology strategy for site-specific labeling of large protein assemblies, we applied single-molecule Förster resonance energy transfer to capture real-time dynamics of the second winged helix domain of the Pol III subunit Rpc34. We find this Rpc34 winged helix fluctuates among discrete positions across the DNA-binding cleft, supporting its role as a dynamically engaged element analogous to cleft-associated stabilizing factors in Pol I and Pol II, with implications for elongation complex organization and potentially rapid re-initiation in Pol III. Our bio-orthogonal labeling approach provides a general framework for probing conformational dynamics in macromolecular machines.

Among the three eukaryotic nuclear RNA polymerases, RNA polymerase III (Pol III) is specialized for synthesizing structural and noncoding RNAs that are short in length but essential for cellular function. In humans, dysregulation of Pol III transcription is linked to a range of diseases, including neurodegenerative disorders and cancer (1–3). Pol III synthesizes short RNAs such as tRNAs, 5S rRNA, U6 snRNA, and 7SL RNA (4, 5), and produces these transcripts at levels exceeding those of all other RNA species combined. This high transcriptional output requires Pol III to undergo rapid and repeated cycles of initiation, termination, and reinitiation, distinguishing it mechanistically from Pol I and Pol II.

Pol III is the largest of the three nuclear RNA polymerases, comprising 17 subunits with a total of ~6,600 amino acids and combined molecular mass of approximately 0.7 MDa. In addition to a conserved Pol II–like core, Pol III contains five additional subunits organized into two Pol III–specific subcomplexes, Rpc82/34/31 and Rpc53/37 (6). These subunits are evolutionarily related to the Pol II initiation factors TFIIE and TFIIF (7), but unlike their Pol II counterparts, they remain stably associated with Pol III throughout the transcription cycle. This organization has led to the proposal that Pol III–specific subunits may play roles beyond initiation; potentially functioning as built-in elongation factors, although direct experimental evidence has remained limited (5).

Within the TFIIE-like subcomplex, Rpc34 (also known as RPC6, RPC39, or POLR3F in humans) is unique in lacking a clear counterpart in the TFIIE heterodimer. Despite its small size, Rpc34 is essential for Pol III transcription. Genetic and biochemical studies have established its roles in promoter opening and in bridging Pol III to the initiation factor TFIIIB (8, 9). These functions are mediated by the N-terminal tandem winged helix (tWH) domain, composed of WH1 and WH2 (10). More recent biochemical analyses have suggested that the Rpc34 tWH domain may also contribute to transcription elongation (11), supporting the view that Rpc34 functions at multiple stages of the transcription cycle.

Earlier photoactivated crosslinking (PA-XL) studies provisionally positioned Rpc34 WH2 above the Pol III active site, spanning the DNA-binding cleft and presumably contacting both the Rpc1 (Rpc160) clamp and the Rpc2 (Rpc128) protrusion domain (12) (*SI Appendix*, Fig. S1). In contrast, cryo-EM structures of the Pol III preinitiation complex (13, 14) revealed Rpc34 WH2 shifted upstream, where it engages TFIIIB and the Rpc2 protrusion domain, consistent with a role in PIC assembly (9). In addition, structural studies of the Pol III–Maf1 complex showed that Maf1 occupies the TFIIIB-binding site and contacts Rpc34 WH2, constraining its mobility and repressing transcription (15). Together, these observations indicate that Rpc34 WH2 can adopt distinct positional states depending on the transcriptional context.

The role of Rpc34 WH2 during transcription elongation, however, remains poorly understood. This is because its density is absent in elongation complex (EC) cryo-EM reconstructions (16) despite major advances in cryo–EM that have yielded high-resolution structures of Pol III transcription complexes. This density absence suggests Rpc34 WH2’s increased mobility upon transition from initiation to elongation, but direct evidence for such dynamics has been lacking. Notably, the corresponding domain of human RPC6/RPC39 is similarly structurally unresolved in human Pol III ECs (17), suggesting that this flexible conformational behavior could be evolutionarily conserved.

**Fig. 1.**
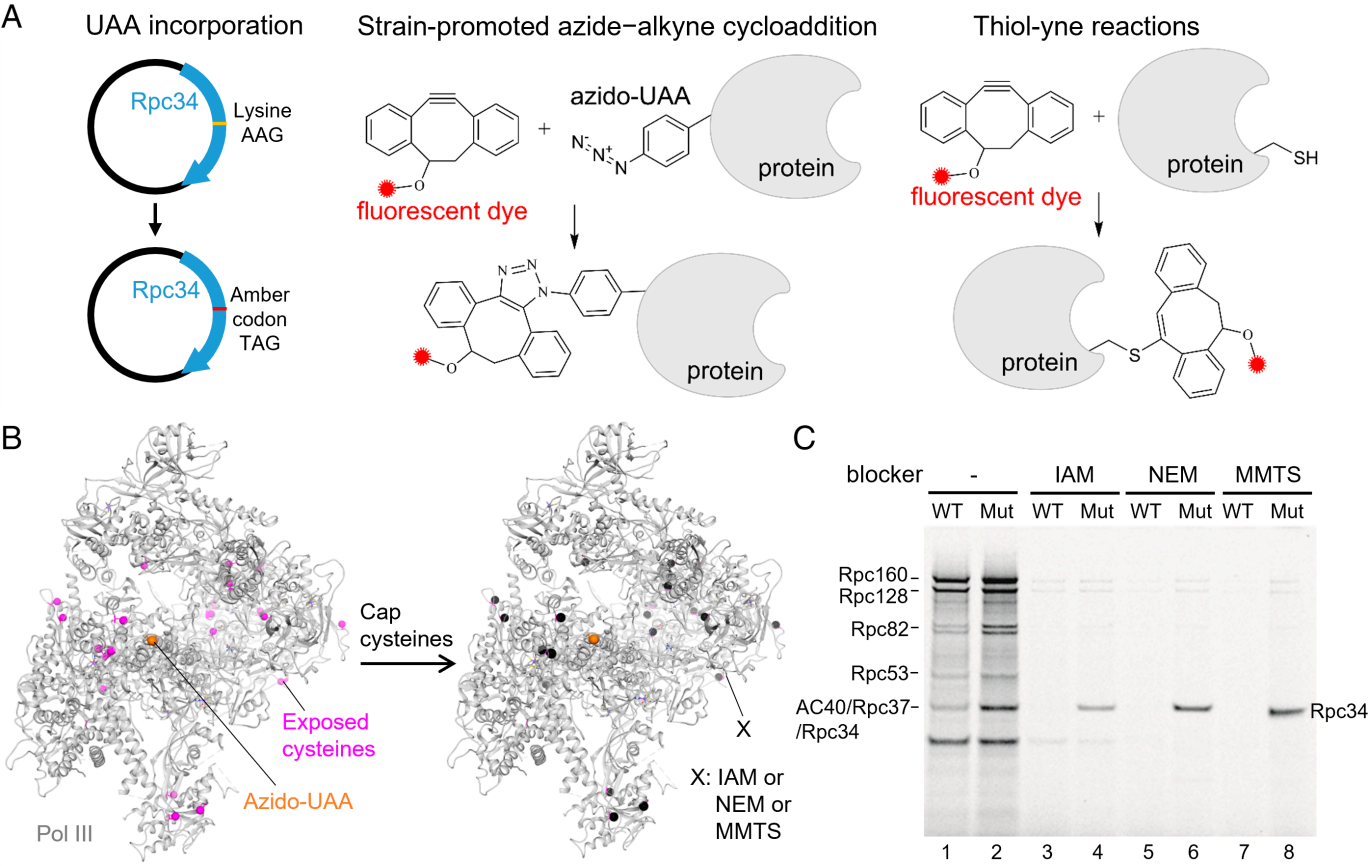
Selective dye labeling of incorporated azido-UAA by capping native cysteines in Pol III. (*A*) *Left*: Schematic representation of UAA incorporation into Rpc34 WH2 in Pol III. The Rpc34/pRS425 plasmid was transformed into a yeast strain lacking chromosomal Rpc34, where a specific codon was mutated to an amber codon for the incorporation of azido-UAA at a designated position. *Middle:* Chemical structures of DIBO (dibenzocyclooctyne, a strained alkyne) and azido-phenylalanine UAA on a protein ready for labeling via strain-promoted azide-alkyne [3 + 2] cycloaddition (SPAAC). *Right*: The reaction of thiol-yne addition, where free thiol groups on cysteines react with the alkyne-dye, leading to nonspecific DIBO dye labeling on Pol III. (*B*) Cysteine Blocking Schematic: this illustration shows that exposed cysteines (magenta spheres) on Pol III can be capped by thiol-reactive molecules (black spheres), inhibiting nonspecific DIBO dye labeling. (*C*) Screening of Thiol Blockers: Thiol-reactive molecules—iodoacetamide (IAM), N-ethylmaleimide (NEM), and S-methyl methanethiosulfonate (MMTS)—were tested at a concentration of 20 mM. The Alexa647 fluorescence image of an SDS-PAGE gel shows that IAM, NEM, and MMTS effectively abolished nonspecific DIBO-Alexa647 labeling of wild-type Pol III and azido-UAA-modified Pol III (see also *SI Appendix*, Fig. S3).

Here, we investigate the conformational dynamics of Rpc34 WH2 in a yeast Pol III EC using single-molecule Förster resonance energy transfer (smFRET), a technique capable of resolving conformational heterogeneity and dynamic interconversions at the level of individual molecules (18). To enable site-specific labeling in the context of the cysteine-rich Pol III complex, we implemented a bio-orthogonal strategy based on azide unnatural amino acid (UAA) (19, 20) incorporation modified from a photoactivated probe UAA system (12, 21) and strain-promoted click chemistry [strain-promoted azide-alkyne [3+2] cycloaddition (SPAAC) innovated by Bertozzi (22)—now most commonly known as “copper-free click reaction”], and developed a simple chemical step (Fig. 1) to suppress previously overlooked off-target labeling (23–25) caused by the cross-reactivity between cyclooctynes and thiols (26, 27). By combining smFRET with nano-positioning system (NPS) triangulation (28, 29), we localize Rpc34 WH2 to multiple preferred positions distributed across the Pol III DNA-binding cleft. These results indicate that Rpc34 WH2 engages in transient and weak interactions with the EC, providing a structural framework for understanding its multifunctional roles during Pol III transcription.

## Results

### Capping Thiols Enables Selective Alkyne–Dye Labeling Via Strain-promoted Click Reaction.

To enable site-specific fluorescent labeling of Pol III, we incorporated a single 4-azido-L-phenylalanine (AzF) residue into Rpc34 using a yeast-based UAA system (Fig. 1*A* and *Materials and Methods*). AzF restored the viability of mutant yeast cells (*SI Appendix*, Fig. S1), and its incorporation did not affect Pol III transcriptional activity, where Pol III complexes labeled with a strained-alkyne fluorophore (DIBO–Alexa647) remained catalytically competent (Fig. 1*C* and *SI Appendix*, Fig. S2).

Unexpectedly, SPAAC resulted in extensive off-target labeling of multiple Pol III subunits lacking AzF (see *lane 1* in Fig. 1*C*), consistent with nonspecific reactions between the strained alkyne and surface-exposed cysteine residues. Consistent with prior reports of thiol–cyclooctyne cross-reactivity (Fig. 1*A*) (26, 27), our precapping cysteine thiols with small molecules (Fig. 1*B*) effectively eliminated nonspecific labeling (lanes 3 to 8 in Fig. 1*C*). Among the reagents tested, methyl-methanethiosulfonate (MMTS) (30) selectively suppressed off-target reactions while preserving efficient labeling of Rpc34, without compromising elongation complex assembly (Fig. 1*C* and *SI Appendix*, Fig. S3). This strategy enabled robust and selective fluorophore conjugation at a single engineered site within the native Pol III complex.

### SmFRET Reveals Discrete and Dynamic Positional States of Rpc34 WH2 in the Pol III EC.

**Fig. 2.**
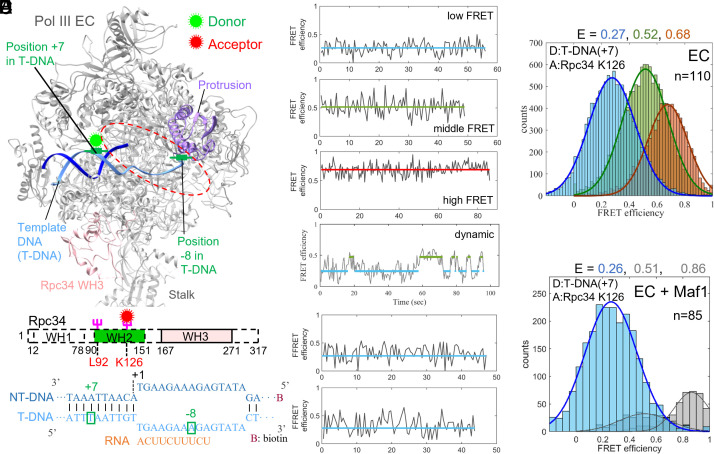
smFRET reveals multiple conformational states of Rpc34 WH2 in the Pol III EC. (*A*) Design of the smFRET labeling scheme based on the yeast Pol III EC structure (PDB: 5fj8). The cryo-EM density corresponding to the Rpc34 tWH domain is absent in this structure. For clarity, Rpc82 and Rpc31 are omitted from the displayed model. The donor dye (TAMRA) is positioned at the +7 site on the template DNA (cyan), and the acceptor dye (Alexa647) is attached to Rpc34 WH2 (e.g., K126). Biotin, denoted by “B,” is at the 5′ end of NT-DNA for immobilization on the microscope slides. The dye linkage chemistry is described in *SI Appendix*, Fig. S5. (*B*) Four representative smFRET time trajectories: three distinct stable low-, middle-, and high-FRET states, and a dynamic FRET-state switching of Rpc34 WH2 in Pol III EC in the absence of Maf1. Note that the dynamic one is the same as in trace 1 in Fig. 3*A*. Horizontal lines indicate mean FRET efficiencies for each state. (*C*) Frame-wise smFRET histogram constructed from all trajectories acquired in the absence of Maf1, resolving three distinct populations that are each well fit by a single Gaussian, with mean FRET efficiencies of 0.27 (low), 0.52 (middle), and 0.68 (high). “E” denotes mean FRET efficiencies, and “n” number of molecules). (*D*) Representative smFRET time trajectories of Pol III EC in the presence of Maf1, showing predominantly single, stable low-FRET behavior. (*E*) Frame-wise smFRET histogram acquired in the presence of Maf1, exhibiting a dominant population centered at a mean FRET efficiency of 0.26.

To probe Rpc34 WH2 dynamics in the Pol III EC using smFRET with a donor-acceptor pair, we labeled Rpc34 at residue K126 with the acceptor dye (Alexa647) and placed the donor dye (TAMRA) at DNA position +7 as a fixed reference point downstream of the active site (Fig. 2*A* and *SI Appendix*, Fig. S4), where the chemical structure of dye linkage is described in *SI Appendix*, Fig. S5. This DNA position is well resolved in Pol III cryo-EM reconstructions (16), providing a stable structural reference. Prior to Pol III measurements, the accuracy of the smFRET setup was validated using a benchmark DNA system, for which measured FRET efficiencies closely matched values predicted by dye-swirling models (*SI Appendix*, Fig. S6) (31, 32).

SmFRET measurements were performed on immobilized, dual-labeled Pol III ECs using a customer-built objective-lens-type total internal reflection fluorescence microscopy with alternating laser excitation (ALEX) (*Materials and Methods*). Analysis of 110 single-molecule time trajectories with clear acceptor photobleaching signatures allowing for extracting absolute FRET efficiencies (33) revealed multiple FRET states (Fig. 2*B* and *SI Appendix*, Fig. S7). Approximately half of the molecules exhibited stable distinct FRET levels predominantly contaminated by shot noise (34), whereas the remainder showed dynamic switching between levels. We noticed that the donor intensities appeared to become “noisier” after the acceptor bleaches. Such an increase in donor intensity fluctuations after acceptor photobleaching is routinely and universally observed in shot-noise-limited single-molecule FRET measurements and arises directly from the √N scaling of photon-counting statistics (“N” stands for number of photons), regardless of the presence or absence of a protein (*SI Appendix*, Fig. S8). Frame-wise histograms constructed from all trajectories resolved three well-separated FRET populations centered at efficiencies of approximately 0.68, 0.52, and 0.27 (Fig. 2*C*). These values were consistent between molecules displaying static and dynamic behavior, indicating the presence of three discrete conformational states rather than a continuum.

To determine whether the observed FRET dynamics primarily reflect motion of Rpc34 WH2, we constrained its mobility by adding excess Maf1, which stabilizes Rpc34 WH2 in a defined position as observed in cryo-EM structures of the Pol III–Maf1 complex (15). Under these conditions, the vast majority of molecules exhibited single-level, stable FRET trajectories (Fig. 2*D*), in marked contrast to the multistate behavior observed in the absence of Maf1. These controls argue against DNA motion as a dominant contributor to the observed FRET dynamics. The mean FRET efficiency obtained in the Maf1-constrained state (E ≈ 0.26; Fig. 2*E*) closely matched the modeled value predicted based on the cryo-EM structure (15) using established Förster parameters of 6 nm for this dye pair (35, 36) (*SI Appendix*, Tables S2 and S3). This agreement demonstrates that the smFRET measurements provide quantitative distance constraints consistent with available structural models.

A small fraction of molecules exhibited higher FRET efficiencies in the presence of Maf1 (Fig. 2*E*), consistent with partial Maf1 dissociation during imaging. To further validate the robustness of these observations, we performed analogous experiments with the acceptor dye positioned at Rpc34 residue L92, also within the WH2. As observed for K126 labeling, Maf1 addition largely eliminated multistate behavior, yielding a mean FRET efficiency (E ≈ 0.53) (*SI Appendix*, Fig. S9) in excellent agreement with corresponding structural modeling (*SI Appendix*, Fig. S10). Moreover, in the absence of Maf1, labeling at L92 similarly exhibited multiple FRET states (*SI Appendix*, Fig. S11).

Together, these results demonstrate that Rpc34 WH2 occupies multiple preferred positional states within the Pol III EC. Based on their relative FRET efficiencies, we designate these states as distal (E ≈ 0.27), middle (E ≈ 0.52), and proximal (E ≈ 0.68) with respect to the DNA(+7) reference site. The existence of discrete, interconverting FRET states indicates that Rpc34 WH2 undergoes dynamic repositioning on Pol III EC, rather than sampling a single static configuration during elongation.

### Dwell-Time Analysis Reveals Characteristic Lifetimes and Ordered Transitions Among Rpc34 WH2 Conformational States.

**Fig. 3.**
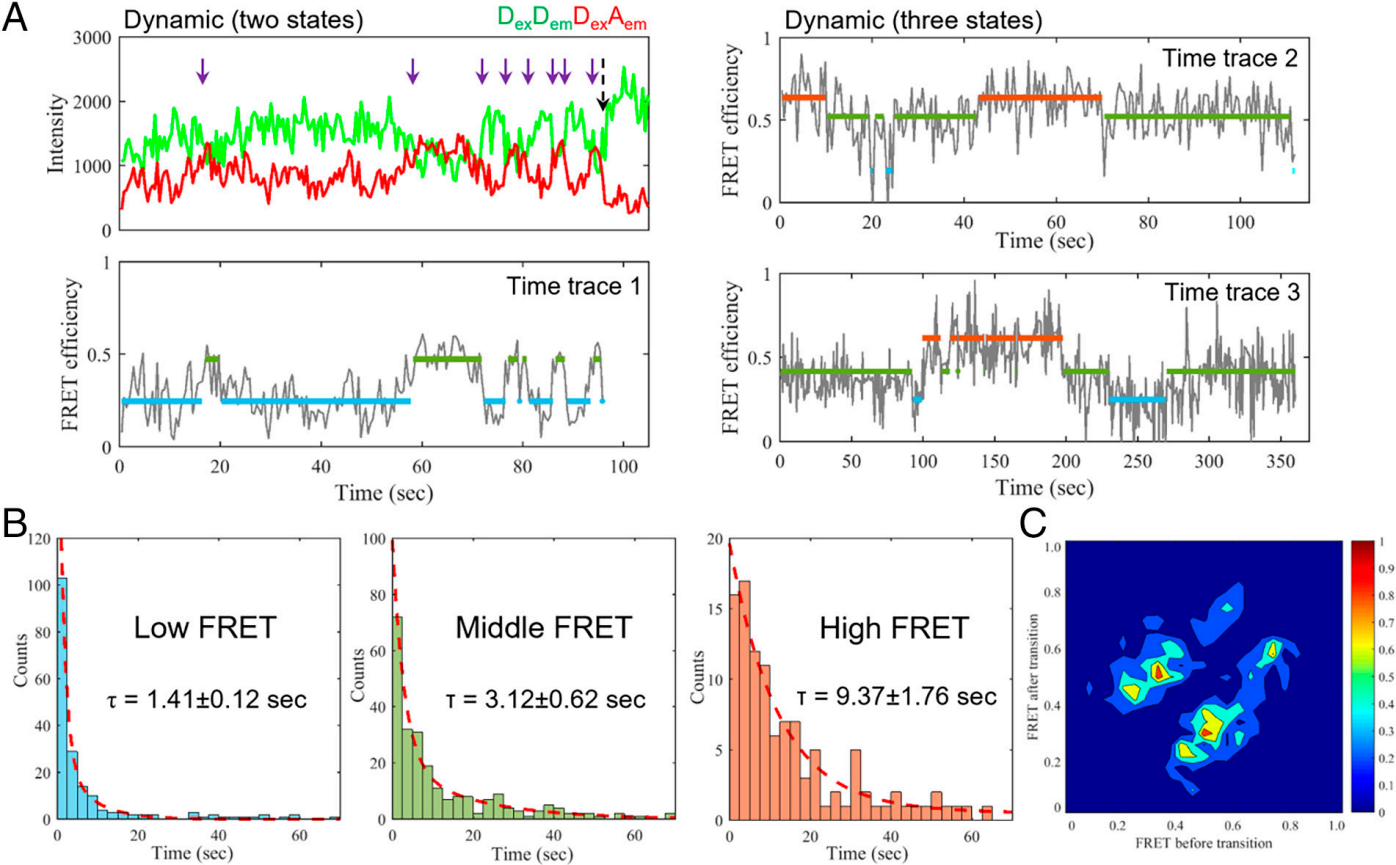
Representative time trajectories of interstate converting FRET and dwell-time analysis for Rpc34 WH2 in Pol III EC. The donor dye (TAMRA) and acceptor dye (Alexa647) were labeled at the +7 position of the template DNA and Rpc34 K126, respectively. (*A*) *Left*: A representative time trajectory exhibiting interstate conversion between two states is shown. The green and red traces represent the donor emission (Dem) and acceptor emission (Aem) intensities upon donor excitation, respectively. Purple-black arrows indicate FRET-associated anticorrelated signals between the donor and acceptor, with the last arrow in black highlighting the anticorrelation event resulting from acceptor photobleaching. The corresponding FRET time traces (gray) were calculated from the donor and acceptor intensity time traces based on individual γ normalization. HMM was applied to these intensity time traces to identify the levels of each FRET state: low (light blue), middle (light green), and high (orange), along with their respective durations. *Right*: Another two representative time trajectories demonstrate interstate conversion among three states, with HMM-extracted FRET levels color-coded accordingly (low: light blue, middle: light green, high: orange). (*B*) The dwell-time histograms for low-, middle-, and high-FRET states are presented, based on HMM results from 62 state-changed traces in one experiment. The average dwell time and SD were calculated from three separate experiments (N = 3: three biological replica) (*SI Appendix*, Fig. S13). (*C*) A transition density plot constructed from 633 transition events pooled from multiple experiments illustrates the major and minor populations of transitions. The major transitions occur between the middle-FRET state and the low-FRET state, while minor transitions occur between the middle-FRET state and the high-FRET state.

SmFRET time trajectories exhibiting interstate switching directly report real-time motion of Rpc34 WH2 within the Pol III EC. To quantify the kinetics of these transitions, we analyzed dynamic trajectories using hidden Markov modeling (HMM) (37), which identifies state transitions and extracts the dwell time spent in each FRET state (*Materials and Methods*). ALEX was used to exclude spurious FRET events arising from acceptor quenching (*SI Appendix*, Fig. S12). HMM analysis of those transitions resolved the same three FRET states (Fig. 3*A*), with mean efficiencies consistent with values obtained by frame-wise histogram analysis (Fig. 2*C*).

Pooling dwell times across molecules yielded distributions that were well described by single-exponential decays (Fig. 3*B*), indicating characteristic lifetimes for each state rather than a broad continuum of timescales. Across three independent biological replicates (*SI Appendix*, Fig. S13), the average lifetimes were 9.37 ± 1.18 s for the high-FRET state, 3.12 ± 0.48 s for the middle-FRET state, and 1.41 ± 0.11 s for the low-FRET state, with the high-FRET state being the most long-lived. It is worthwhile to note that transitions faster than the frame rate would be underrepresented, and biasing against those discrete states.

To examine transition connectivity among states, we constructed two-dimensional transition histograms (Fig. 3*C*). Direct transitions between the low- and high-FRET states were not found, whereas transitions predominantly proceeded through the middle-FRET state. Consistent with this observation, separating middle-state dwell times into middle-to-high and middle-to-low transitions (*SI Appendix*, Fig. S14) allowed estimation of state-specific transition rates (Fig. 5). Together, these results indicate that Rpc34 WH2 transitions among discrete conformational states likely through an ordered kinetic pathway, with the middle-FRET state serving as a central intermediate.

### Nano-Positioning Using smFRET Data Identifies Rpc34 WH2 Preferred Positions.

The high, middle, and low FRET states observed for the acceptor-labeled Rpc34 K126 correspond to the proximal, middle, and distal sites relative to DNA(+7). To determine the spatial location of these sites within the Pol III EC, we employed NPS triangulation analysis (28, 29). This medium-resolution method determines the position of an “antenna” (the probe) relative to fixed “satellites” (known coordinates) based on “interdye distances” converted from FRET efficiencies. NPS has previously proven effective in elucidating mobile elements within Pol II complexes (36).

**Fig. 4.**
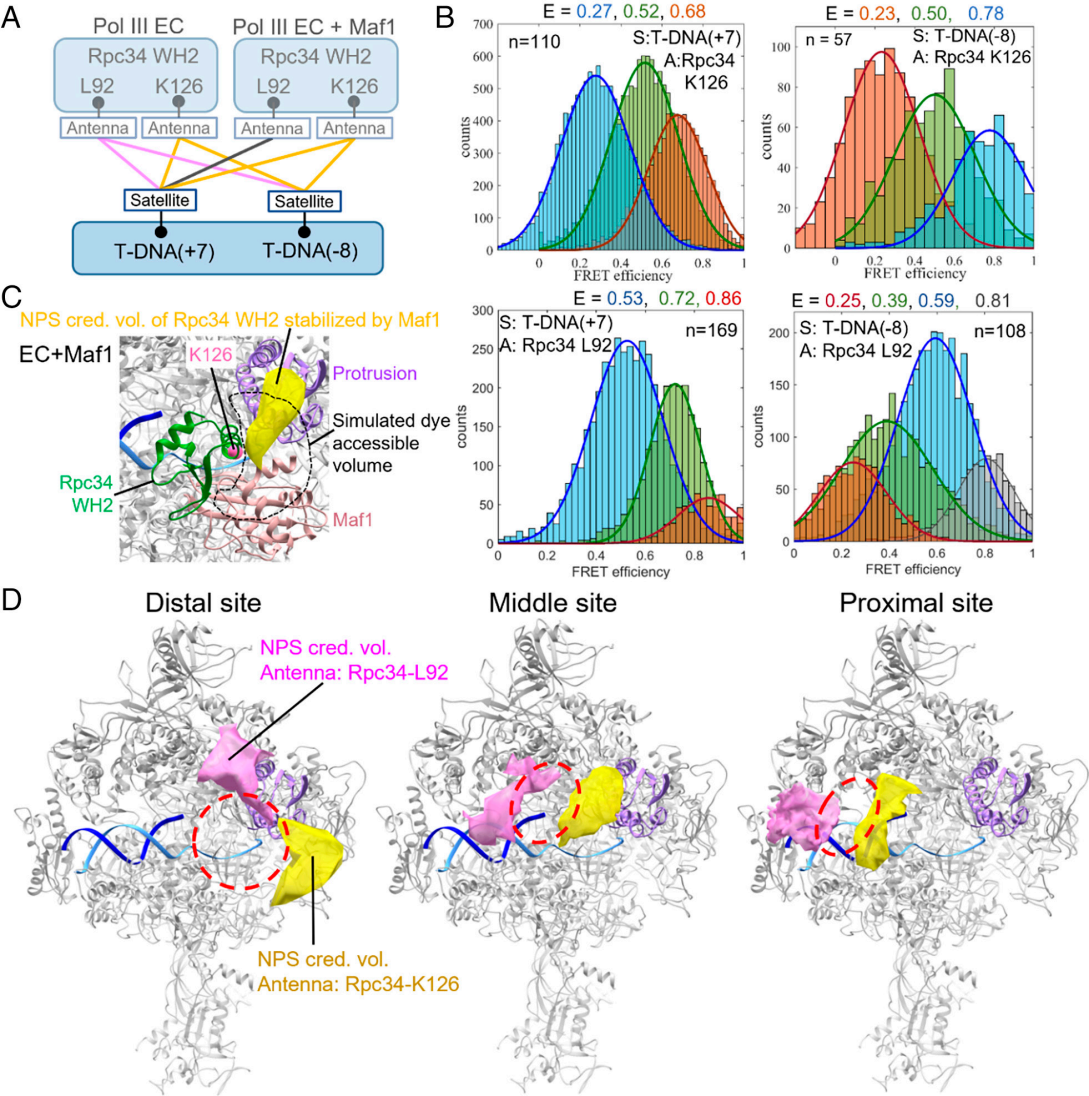
Nano-positioning (NPS) localization of Rpc34 WH2 in Pol III EC. (*A*) Schematic representation of the FRET network with the satellite and antenna positions for spatial mapping by NPS. (*B*) The four FRET efficiency histograms provide mean FRET efficiency of satellite-antenna (S/A): +7/126, −8/126, +7/92, and −8/92, for localizing Rpc34 K126 and L92, with +7/126 indicating the satellite dye (TAMRA) labeled at the +7 position in the template DNA, and the antenna dye (Alexa647) labeled at Rpc34 K126. The colors in the histogram for +7/126—light blue, light green, and orange—correspond to the low-, middle-, and high-FRET states, with E, S, A, and n representing the mean FRET efficiencies, satellite, antenna, and the number of molecules. (*C*) NPS localization of Maf1-stabilized Rpc34 WH2: this control localizes Rpc34 WH2 in Pol III EC at the “distal” site (Fig. 2*C* and *SI Appendix*, Fig. S9*B*), where the NPS credible volume (yellow) is found to have good overlap with the simulated dye accessible volume (dashed area) (*SI Appendix*, Fig. S10). (*D*) NPS-derived credible volumes for Rpc34 K126 (yellow) and L92 (pink), shown in Pol III EC structure. Three sites, flanked by a pair of volumes, are identified—the distal site (*Left*) is near protrusion, the middle site (*Center*), and the proximal site (*Right*) occupy the middle and downstream regions of the DNA-binding cleft.

To localize an antenna, such as the dye at Rpc34 K126, a minimum of two satellites is required. To roughly define the position and orientation of the entire Rpc34 WH2 domain, we utilized a second antenna (L92) located on the side of Rpc34 WH2 domain opposite to K126. Our network, as illustrated in Fig. 4*A*, utilized two satellites: Satellite_1 at DNA(+7) (downstream DNA) and Satellite_2 at DNA(−8) (upstream DNA). We adopted a shorthand notation (S/A) to describe these pairs; for example, +7/126 denotes the FRET pair between the DNA(+7) donor and the Rpc34-WH2’s K126 acceptor.

We first validated the precision of this NPS framework using the Maf1-restrained Rpc34 WH2 complex as a control. By measuring FRET for the pairs of +7/126 (0.26) (Fig. 2*C*) and −8/126 (0.55) in the presence of Maf1 (*SI Appendix*, Fig. S9), we generated a credible volume for the K126 dye and found it closely matched that expected from that simulated for a dye attached to K126 in Maf1-stabilized Rpc34 WH2 (*SI Appendix*, Fig. S10). Notably, this experimentally determined volume had reasonable overlap with the simulation results, showing a spatial mismatch of less than 1 nm (6.9 Å) when the dye volume centers were compared (Fig. 4*C*), thereby supporting the system’s precision.

We then applied this validated network to the dynamic Pol III EC in the absence of Maf1 (Fig. 4*A*). Multiple smFRET efficiencies observed from both satellites (Fig. 4*B*) followed by NPS analysis identified three distinct combinations for K126 (+7/126 and −8/126) that are meaningful: (+7/126 = 0.27, −8/126 = 0.78), (+7/126 = 0.52, −8/126 = 0.50), and (+7/126 = 0.68, −8/126 = 0.23), yielding three credible volumes (yellow in Fig. 4*D*). We repeated this for the L92 antenna, which also yielded three distinct credible volumes (pink in Fig. 4*D*) based on the combinations (+7/92 = 0.53, −8/92 = 0.59), (+7/92 = 0.72, −8/92 = 0.39), and (+7/92 = 0.86, −8/92 = 0.25) (*SI Appendix*, Fig. S5). By integrating the spatial information from credible volumes of both K126 and L92, we localized three positions for Rpc34 WH2 (*SI Appendix*, Fig. S15): i) the distal site, which is located upstream of the DNA-binding cleft, adjacent to the protrusion domain, largely overlapping with that in previously reported PIC structures (13, 14); ii) the middle site, which is localized within the central region of the DNA-binding cleft; and iii) the proximal site, which is situated in the downstream region of the cleft.

## Discussion

In this study, we combined single-molecule FRET, nano-positioning (NPS) triangulation, and structural modeling to determine the dynamics and position of the Rpc34 WH2 domain within the Pol III EC. This strategy enabled us to recover the positional information that is consistently missing from high-resolution Pol III EC cryo-EM structures (16, 17). The observation of discrete FRET states—rather than a continuum expected from a WH2 domain that either contacts only DNA or tethers without stable anchoring—argues that Rpc34 WH2 engages Pol III EC, but through weak, transient interactions, in part mediated by dsDNA (38, 39), and in part by protein–protein contacts between Rpc34 WH2 and Pol III core (12–14). The precision of our FRET system and the well-separated FRET-derived “distances” permit unambiguous and accurate NPS localization of those sites in Pol III EC.

**Fig. 5.**
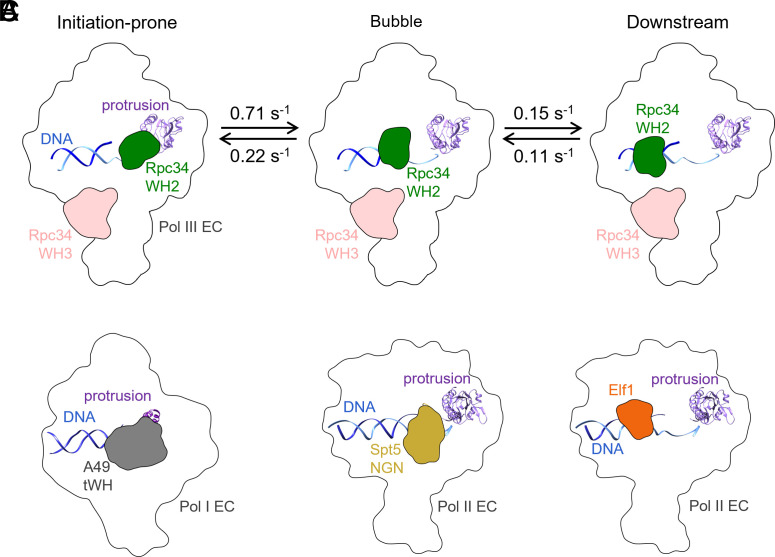
Rpc34 WH2 positioning in Pol III EC and comparison with Pol I and Pol II ECs. (*A*) The three preferred positions of Rpc34 WH2 of TFIIE-like subcomplex in Pol III EC (PDB: 5fj8), upstream (initiation-related), middle (transcription bubble related), and downstream, are identified using the NPS credible dye volumes of L92 and K126 that flank Rpc34 WH2 (Fig. 4*D*). The transition rates among these configurations are obtained from the dwell-time analysis of state-change trajectories of those Pol III ECs that actively switch configurations during the imaging experiments (*SI Appendix*, Fig. S12). The linker connecting Rpc34 WH2 and Pol III-stalk anchored Rpc34 WH3 comprises 16 amino acids, and it can well accommodate the distances of 44, 47, and 53 Å between these two domains in three distinct configurations. Notably, the dwell-times of Rpc34 WH2 in Pol III EC are apparently anticorrelated with the distances between Rpc34 WH2 and WH3. (*B*) Pol I EC (PDB: 5m64) in a configuration of which its DNA-binding cleft is covered by Rpa49 tWH in the TFIIF-like subcomplex (41). (*C*) Two Pol II ECs. In one complex, the downstream DNA-binding cleft is covered by elongation factor Elf1 (42) (PDB: 5xog); in another complex the upstream cleft is covered by the NGN domain of elongation factor Spt5 (42) (PDB: 5xon).

These analyses identified three preferred positions for Rpc34 WH2—proximal, middle, and distal—distributed across the DNA-binding cleft (Fig. 5*A*). Strikingly, these locations map closely onto the Pol III clamp head, the clamp coiled-coil domain, and the region adjacent to the protrusion domain, respectively. These areas were previously identified by crosslinking as Rpc34–Pol III contact sites (12). However, that earlier study (12) could not resolve which Rpc34 domain(s) were involved or whether multiple conformations existed. This was due to two primary factors: i) the PA-XL probe was placed on Rpc1 rather than Rpc34 (12), and ii) the lysine–lysine crosslinking MS analysis, used to complement the PA-XL analysis (12), generally struggles with conflicting crosslinks—for instance, when an identical region of Rpc34 crosslinked to distinct Rpc1 sites—such as the reported clamp head (Ala206–Pro209) and the clamp coiled-coil (Asp304–Ile307) (12). Notably, this limitation has been addressed by the recent Bayesian-based MS analysis developed by Nilges and colleagues (40), which allows for interpreting conflicting crosslinks as evidence of alternative conformations. The high degree of agreement between these historical crosslinks (12) and our smFRET/NPS results provides independent validation for the accuracy of our positional measurements. Compared to crosslinking methods, smFRET has advantages in resolving molecular heterogeneity and capturing temporal behavior. We observed characteristic state-specific kinetics and found that transitions consistently pass through the middle state, which corresponds to an area overlapping with the clamp coiled-coil domain (Figs. 4*D* and 5). These conformational transitions, permitted by a 16 a.a. linker connecting Rpc34’s WH2 to its WH3 stably associated with Pol III’s stalk (Fig. 5*A*), reflect that Rpc34 WH2 can sample different sites during Pol III elongation, with implications for distinct functions, where the site-sampling seems to follow structured, not random, fluctuations. Below we discuss the potential functions of Rpc34 WH2 at each site.

The observed distribution of Rpc34 WH2 positions across the DNA-binding cleft aligns well with previous in vitro evidence implicating Rpc34 tWH in Pol III elongation (11). These observations reveal Rpc34 WH2 as a mobile DNA-engaging module that dynamically explores the Pol III cleft. This DNA-engaging capability is perhaps functionally analogous to cleft-associated regulatory modules that stabilize ECs in Pol I and Pol II, the evolutionary ancestor or parallel of Pol III (43, 44). Rpc34 tWH is homologously related to the tWH domain of Rpa49 in the TFIIF-like subcomplex of Pol I and to the second subunit of the TFIIE initiation factor in the Pol II system. Beyond sequence similarity, Rpc34 tWH and Rpa49 tWH share two notable features: both possess intrinsic DNA-binding capacity (38, 39, 45) and are highly mobile through flexible linkers connecting them to their anchoring modules. In Pol I, this mobility becomes evident during initiation, where cryo-EM structures reveal Rpa49 tWH occupying distinct positions across intermediates of the transcription complex (41). During elongation, its position becomes difficult to resolve structurally (42), indicating substantial conformational flexibility. Crosslinking studies have placed Rpa49 tWH above the upstream DNA-binding cleft (46) (Fig. 5*B*), consistent with the position resolved by cryo-EM heterogeneous structure analysis (42) and a transient interaction with the melted DNA that helps stabilize the EC and its role in promoting processivity (45). In the Pol II system, although TFIIE dissociates upon transition to elongation, its position near the upstream DNA-binding cleft is occupied by the conserved elongation factor Spt5. The NGN domain of Spt5 engages the DNA fork in this region, contributing to EC stability and processivity (47) (Fig. 5*C*). Downstream DNA engagement is further supported by Elf1, a small transcription factor that helps maintain DNA positioning within the cleft during elongation (47). Comparison of Rpc34 WH2 positions with these closely or distantly related cleft-associated elements in Pol I and Pol II with established structure and function relationship strongly suggests that Rpc34 WH2 may perform similar roles during Pol III elongation. When positioned near the middle or upstream regions of the cleft, Rpc34 WH2 could interact with the melted DNA to stabilize the transcription bubble and prevent reannealing, whereas positioning over the downstream region may allow it to act as a dynamic cover that helps secure the DNA template within the cleft. Such interactions could contribute to EC stability or enhance Pol III processivity when the enzyme encounters barriers such as nucleosomes or RNA secondary structures, despite the relatively short transcription units typically transcribed by Pol III (13, 16, 48). Together, these comparisons suggest that mobile cleft-associated DNA-engaging elements represent a conserved strategy by which eukaryotic RNA polymerases stabilize ECs.

Surprisingly, the upstream position overlaps with the WH2 location observed in the preinitiation complex, indicating positional continuity across transcriptional states, and raises the possibility that WH2 spatial fluctuations in the EC may allow Pol III to adopt configurations poised for rapid re-engagement with TFIIIB, potentially facilitating efficient reinitiation—a distinctive feature of the Pol III transcription system. More broadly, these observations lead to a postulate that Rpc34 WH2 mobility may function as a regulatory element linking different stages of the Pol III transcription cycle, enabling coordinated transitions between initiation, elongation, and rapid reinitiation. While direct effects on elongation stability or reinitiation were not examined here, these structural observations provide a framework for understanding how Rpc34 WH2 dynamics may be integrated into the rapid transcription cycle of Pol III.

Besides revealing the conformation dynamics of Rpc34 WH2 in Pol III EC, we uncovered and solved a commonly overlooked source of off-target labeling in SPAAC (commonly known as Bertozzi copper-free click reaction) (22): cross-reactivity between alkynes and thiols. This issue severely compromises selectivity when labeling large native complexes containing many reactive cysteines. By implementing a simple thiol-capping strategy, we restored selective reactivity toward the engineered azido residue in Pol III. Although Staudinger ligation is free from this cross-activity issue and has been therefore used to label azido-modified proteins (49, 50), its applicability is now limited by the seemingly diminishing availability of suitable reagents. Our approach thus reestablishes SPAAC as a practical and robust route for site-specific labeling of large native protein assemblies for smFRET-based dynamic structural studies (51). During method development, we noted that certain thiol-capping reagents (e.g., MMTS) can, under some conditions, may modestly alter the distribution of elongation products in bulk transcription assays. While this effect did not preclude formation of functional ECs for smFRET measurements, it suggests that the choice and extent of thiol modification should be carefully optimized when applying this strategy to enzymatic systems.

Taken together, our results reveal that Rpc34 WH2, despite its small size, is a dynamically positioned and multifunctional element within the Pol III EC. By visualizing its real-time conformational behavior, we provide molecular insights into how this domain may contribute to DNA stabilization, bubble maintenance, and potentially reinitiation. This work enables real-time visualization of auxiliary-subunit dynamics within the Pol III core, and establishes a versatile labeling framework that should benefit smFRET analysis of other large macromolecular machines.

## Materials and Methods

Detailed descriptions are available in *SI Appendix*, *SI Materials and Methods* for plasmids and yeast strains, protein purification, Pol III labeling, EC formation and in vitro transcription, smFRET system with ALEX, swirling dye volume modeling for the predicted FRET efficiencies, smFRET movie data reduction and FRET histogram analysis, FRET state dwell-time analysis with exponential fitting, and credible dye volume localization using nanopositioning triangulation analysis.

## Supplementary Material

Appendix 01 (PDF)

## Data Availability

All data needed to evaluate the conclusions in the paper are present in the paper and/or the Supplementary Information. The associated raw and processed data are publicly available via Zenodo (https://doi.org/10.5281/zenodo.20741879) (52). Custom MATLAB and Python scripts used for wide-field smFRET data analysis are available on GitHub (https://github.com/weihaulab/wf-smfret-pipeline.git) (53).
