# Supplementary material for: Dynamic positioning of Rpc34 winged helix in RNA polymerase III elongation complex for its stability with implications for reinitiation
Source: Proc Natl Acad Sci U S A. 2026 Jun 29;123(27):e2601775123. doi: 10.1073/pnas.2601775123 (PMC13343003; doi:10.1073/pnas.2601775123)
Supplement: Supplementary file 1 — Appendix 01 (PDF) [file pnas.2601775123.sapp.pdf]

Supplementary Information

for

Dynamic positioning of Rpc34 winged-helix in RNA polymerase III elongation complex for its stability with implications to re-initiation

Jheng-Syong Wu[1], Yu-Chun Lin[2], Yi-Yu Wei[2], Hsin-Hung Lin[1,3], Yang-Chih Liu[1], Jen-Wei Chang[1,†], I-Ping Tu[3], Hung-Ta Chen[2,*] and Wei-Hau Chang[1,4,5,*]

[1] Institute of Chemistry, Academia Sinica, Taipei, 115, Taiwan

[2] Institute of Molecular Biology, Academia Sinica, Taipei, 115, Taiwan

[3] Institute of Statistical Science, Academia Sinica, Taipei, 115, Taiwan

[4] Genomic Research Center, Academia Sinica, Taipei, 115, Taiwan

[5] Institute of Physics, Academia Sinica, Taipei, 115, Taiwan

[*] Correspondence should be addressed to Hung-Ta Chen. Email: htchen012@gate.sinica.edu.tw, or Wei-Hau Chang. Email: weihau@gate.sinica.edu.tw

[†] Present Address: Development Center for Biotechnology, Taipei, 115, Taiwan

This Supplementary Information file contains

SI Materials and Methods

15 Supplementary Figures

4 Supplementary Tables

SI Materials and Methods

Plasmids, yeast strains and cell growth

Incorporation of UAA into yeast was achieved as described previously[1,2]. In brief, to generate a yeast strain with UAA incorporated into Rpc34, the coding gene sequence of Rpc34 with a V5 epitope tag at the N terminus was first cloned into a 2-micron vector pRS425 (LEU2+) (Fig. 1a). The TAG (amber) nonsense codon was introduced into the Rpc34/pRS425 plasmid by in vitro mutagenesis at a designated position for expressing the UAA, and a series of single UAA Rpc34 mutants at different positons were thereby created. To charge the tRNA with azido-UAA (4-Azido-L-phenylalanine, AzF), the EcTyrRS gene coded by the plasmid pLH157 ($tRNA_{CUA}$/BPA-tRNA synthetase) was modified to become pLH157-AzF ($tRNA_{CUA}$/AzF-tRNA synthetase plasmid). Specifically, the EcTyrRS gene was mutated at the following positions: Tyr37Leu, Asp182Ser, Phe183Met, and Leu186Ala according to that previously described[1]. The Rpc34 mutant/pRS425 plasmid and pLH157-AzF were then co-transfected into the *Saccharomyces cerevisiae* YLy3 yeast strain [MATα ade2::his3G his3Δ200 leu2Δ met15Δ lys2Δ trp1Δ63 ura3Δ (rpc34::KanMX4) Rpc34/pRS316 (Ura3+)] modified from the BY4705 strain. In this yeast strain, the chromosomal Rpc34 gene was first disrupted by a KanMX gene cassette resistant to the drug G418 with the cell viability supported by Rpc34 gene in the pRS316 (Ura3+) plasmid as Rpc34 is an essential. Next, rpc34 amber mutation carried by the pRS425 (Leu2+）plasmid was introduced into a haploid strain, and plasmid shuffling strategy with 5FOA dropout procedure was used to replace the Rpc34/pRS316 (Ura3+). Yeast plate assays were performed—cell viability of a mutant strain on a 5FOA plate ensured that Rpc34/pRS316 (Ura3+) had been eliminated through plasmid shuttling, while that of the strain on a plate with the supplement of azido-L-phenylalanine (AzF)

indicated successful nonsense suppression. Furthermore, non-viability of the mutant strain on a plate with synthetic complete lacking Uracil (GC-Ura) was indicative of the complete loss of the Rpc34/pRS316 (Ura3+) plasmid.

Protein purification

The Pol III was purified by means of the tandem affinity purification (TAP) strategy with a custom-designed TAP tag (FLAG3-His6-TEV cleavage sequence-protein A) fused to the C terminus of the Rpc2 (Rpc128) as described previously[3]. In brief, yeast cell cultures were grown in YPD medium with 0.2 mM AzF to OD = 2.0 and then harvested. Cells from 6-liter cell cultures were pooled and re-suspended in the TAP-tag purification buffer containing 40 mM HEPES (pH 7.5), 350 mM NaCl, 10% glycerol, 0.1% Tween-20, 0.5 mM ethylenediaminetetraacetic acid (EDTA) and 1x protease inhibitors (1 μM pepstatin A, 1 mM phenylmethylsulfonyl fluoride (PMSF), 2.58 mM benzamidine and 0.7 mM leupeptin) and then lysed by glass bead beating. The cell lysate was centrifuged at 5,000 rpm with a JLA-8.1 rotor (Beckman Coulter) for 10 min, and ultra-centrifuged at 35,000 rpm with a Ti-45 rotor (Beckman Coulter) for 1 hour to collect the clarified lysate. The resulting supernatant was incubated overnight with 2 ml IgG-Sepharose resins (GE Healthcare) at 4℃. Subsequently, the bound proteins on the resins were washed once with 50 ml TAP-tag purification buffer and then re-suspended in 1 mL TEV cleavage buffer containing 10 mM Tris (pH 8.0), 150 mM NaCl, 10% glycerol, 0.1% NP-40, 0.5 mM EDTA, supplemented with 50 μg TEV protease and digestion was conducted overnight at 4°C. The eluted protein was verified by Coomassie-blue stained SDS-PAGE and Western blotting for the FLAG and V5 tags, aliquoted and stored at – 80 ℃.

To express *Saccharomyces cerevisiae* Maf1 in *E. coli*, the gene was codon optimized

and synthesized in a pUC57 vector (GenScript Biotech.), and then sub-cloned into pDuet2 vector with the BamHI and XhoI restriction sites to produce a pDuet2-Maf1 plasmid. In this plasmid, the N-terminus of Maf1 has a His6-HA-SUMO tag and the C-terminus a TAP tag (FLAG-Twin Strep Tag with a HRV3C cleavage site in between to facilitate purification. For over-expression of this recombinant Maf1 using *E. coli*, this plasmid was transformed into Rosetta 2(DE3) competent cells (Merck/YB Biotech). Cells were first cultured in 2-liter LB medium containing 30 μg/ml chloramphenicol and 100 μg/ml Ampicillin at 37℃ till OD_{600} reached 0.4 - 0.5, and the protein over-expression was induced with 0.1 mM IPTG overnight at 18℃. The cells were harvested by centrifugation with a JLA 8.1 rotor with 6,545 g for 30 minutes, and re-suspended in lysis buffer containing 1x PBS (pH 8.0), 1x protease inhibitor (made from 50x Cocktail protease inhibitor, Roche), 5mM beta-mercaptoethanol, and then lysed by the constant cell disruption system (TS 2.2Kw, Constant Cell Disruption System). The whole cell extract was ultra-centrifuged at 36,000 rpm to achieve 188,000 g with a Ti-45 rotor for 30 minutes at 4℃ to remove cell debris. The collected supernatant of 50 ml was first incubated with SUMO protease (ThermoFisher) overnight at 4℃ to digest the SUMO tag, then mixed with 0.2 ml 50 % slurry of Strep-Tactin resin (Cytiva) and incubated at 4℃ for 2 hours. After batch washing the Strep-Tactin resins by 100 column volume of “strep-wash” buffer (25 mM Tris-HCl pH 8.0, 500 mM NaCl, 1% NP-40 and 5 mM beta-mercaptoethanol) and equilibrating with an elution buffer (25mM Tris-HCl pH 8.0, 150 mM NaCl, 5% glycerol), the resins was incubated with the elution buffer supplemented with 100 μl of HRV3C with 1mM DTT for overnight at 4℃. Maf1 protein was eluted as fractions with 0.05 ml step; the fractions were further incubated with NTA resins for cleaning the HRV3C protease, examined by SDS-PAGE gel with Coomassie-Blue stain, and verified by MALDI-MS analysis. Two fractions with

highest concentrations were pooled and exchanged to a storage buffer the same as elution buffer except 1mM DDT replaced by 10 mM TCEP, aliquoted and stored at – 80 ℃.

Pol III DIBO-labelling, Pol III EC formation, and RNA extension assay

To form Pol III EC, purified Pol III was incubated with a DNA/RNA oligonucleotide scaffold that contained 10-nucleotide (nt) RNA, where the donor dye of TAMRA was placed on the template DNA (T-DNA), and biotin on the non-template DNA (NT-DNA). The incubation for Pol III-EC was performed in buffer (100 mM HEPES pH 7.9, 400 mM KCl, 10% glycerol, 25 mM $MgCl_2$, 5 mM EDTA). The oligonucleotide sequences and modifications are: (i) template strand with TAMRA dye labelling at the +7 position (5'-CATAAAAAACCCAAAAAAAGAGAGTATT-TAMRA dye-AATTGTTGAAGAAAGAGTATACTACATA); (ii) non-template strand (5'-biotin-TATGTAGATATGAGAAAGAAGTACAATTAAATACTCTCTTTTTTTG GGTTTTTTATG); (iii) RNA strand (5'-UCUUUCUUCA-3'). The three oligonucleotide strands were mixed and annealed to form this DNA/RNA scaffold that contained a 15-nucleotide bubble. An RNA primer extension assay of Pol III EC was performed to evaluate the impact of DIBO-dye labelling or MMTS on the elongation activity of Pol III. DIBO-labelled wild-type Pol III, MMTS-treated Pol III, and DIBO-labeled AzF-substituted Pol III were compared in the contest. DNA/RNA scaffold used in the test included non-dye-labeled and dye-DNA (Supplementary Fig. 2). After Pol III EC formation, free sulfhydryls of cysteines in the Pol III EC were capped with 0.2 mM N-ethylmaleimide (NEM) or 20 μM methyl-methanethiosulfonate (MMTS) as those concentrations were found to not to interfere with formation of Pol III EC, where high concentrations of NEM or MMTS

did interfere with formation of the Pol III EC as evidenced by disappearance of the respective band (~720 kD) in the native gel (Supplementary Fig. 3). Subsequently, 10 μM Alexa647-DIBO (C10408, Thermo-Fisher) was added into 3 μg of Pol III EC to specifically label AzF by copper-free click chemistry reaction at 4 ℃ overnight. Following the reaction, a desalting spin column (Roche) was used to remove excessive dye-DIBO reagent. Dye labelling was verified by fluorescence imaging of the SDS-PAGE gel, and EC formation was verified by fluorescence and Coomassie-blue-stained imaging of native PAGE (Thermo Fisher) (Supplementary Fig. 3).

SmFRET experiments with TIRF system with alternating laser excitation

Our smFRET experiments and analytical methodology were conducted as described previously[4]. In brief, approximately 100 pM Pol III EC labeled with the donor-acceptor dye pair was immobilized on a clean cover-glass that was surface-coated with Neutravidin (Thermo Fisher) and biotin-PEG (Laysan Bio) in a sample chamber[5]. The oxygen scavenger imaging buffer (175 nM protocatechuate-3,4-dioxygenase, 7.8 mM protocatechuic acid, 2 mM Trolox, 50 mM Tris:HCl pH7.8, 150 mM NaCl, 1 mM PMSF and 0.1 mg/ml BSA) was injected into the chamber to increase fluorophore photo-stability[6,7]. Wide-field fluorescence images were acquired using a custom-built dual-view total internal reflection fluorescence (TIRF) microscope in which a 532 nm-wavelength laser (DPGL-2100, Photop Technologies) and a 638 nm-wavelength laser (LuxX 638–100, Omicron) were used to excite the donor and acceptor dyes; an oil-immersion objective (UPLSAPO100XO, Olympus, NA 1.49) was used to focus the laser beam onto the sample and collect fluorescence. The dichromatic mirror (FF640-FDi01, Semrock) separated the collected fluorescence photons into the donor and the acceptor emission arms, with

the two different color images projected onto different areas of the EMCCD camera (DV887DCS-BV, Andor Technology). The movies were captured with a frame rate of 5fps, namely 200 milliseconds per frame. In addition, we implemented an alternating laser excitation (ALEX)[8] scheme using a shutter system (LS3ZM2, Uniblitz Electronic) and relay circuit for switching between the excitation of the donor and that of the acceptor as described[9]. ALEX imaging filters out the time periods when the donor or acceptor is absent due to switching to unwanted photo-physical states.

Simulation of the swirling dye volume and predicted FRET efficiencies

In order to predict the FRET efficiencies anticipated from the positions of Rpc34 WH2 in different existing structural models, we computationally placed the acceptor at the targeted amino acid in Rpc34 WH2 based on a structural model and the donor dye at a site of DNA modeled into it. The modeling of DNA into a structural model was performed by aligning a particular structural model against Pol III EC (PDB: 5fj8) based on Rpc 160 (Rpc1) (Supplementary Fig. 4). Accessible volumes of swirling dyes were simulated by FPS software[10] based on donor dye at DNA and acceptor dye at Rpc34 WH2 with designated dye parameters (size of dye and the length and width of linker) (Supplementary Table 2). The distances R_{MP} between the mean dye positions of two accessible volumes of FRET pair in different models were obtained. We calculated the predicted FRET efficiencies for different models based on the mean dye distances and the Förster radius of dye pair (Supplementary Table 3 & 4). In addition, in the simulation of dye accessible volume attached to a benchmark DNA[11] where the two nucleotides labeled by dye were separated by 15 bp. (Supplementary Fig. 6), the double-strand DNA model was built using the web server of Web 3DNA[12]. Our smFRET measurement of a double-labelled benchmark DNA[11] demonstrates the precision of our system (Supplementary Fig. 6).

SmFRET data analysis and dwell-time analysis and exponential fitting

We used a custom-built two-channel TIRF system to record fluorescence images of the donor and acceptor[4]. Fluorescence movie data were analyzed for identifying the co-localized donor/acceptor pair and extracting the donor and acceptor intensities as time trajectories using customer-built algorithms and programs[13]. The extracted signal intensities of the donor and acceptor were used to calculate FRET efficiencies according to the well-known equation:

$$E = \frac{I_A - \beta I_D}{\left(\left(I_A - \beta I_D\right) + \gamma I_D\right)}$$

as described[14] and according to the method of individual γ normalization[14]. I_A and I_D represent the measured intensities of the acceptor and donor, respectively; βI_D corrects for leakage of donor emission into the acceptor channel, and γ corrects for the difference in detection efficiency and quantum yield between the donor and acceptor. We adopted acceptor-photo-bleaching FRET[14] to validate each candidate FRET trace and also to obtain the absolute FRET efficiencies. This method assesses a time trace based on anti-correlated donor/acceptor signals at the time-point when the acceptor is photo-bleached. Using the stringent criterion of acceptor-photo-bleaching[14], we could screen candidate smFRET traces selected based on donor/acceptor co-localization. Typically, we obtained one to two authentic smFRET traces from each movie. Moreover, considering that the acceptor may not always be active in the time trace of a single molecule, we excited both channels using ALEX to continuously survey the presence of an acceptor so as to detect the period when it is not in the active state, namely in the dark state, prior to photo-bleaching. Using this approach, we filtered out those artefact traces due to acceptor quenching not due to FRET. We used the frame-wise FRET efficiency to build a histogram and fitted it using multiple Gaussian

distributions for extracting the mean FRET efficiencies. Alternatively, the mean FRET efficiencies were obtained from stationary traces by smoothing a trace using a denoising algorithm using wavelet analysis and Bayesian inference[15], and the mean FRET efficiencies were used to categorize a stationary trace. A frame-wise FRET efficiency histogram was built from time traces in a category, and was fitted with single Gaussian distribution for extracting the mean efficiency. These two approaches gave identical results in terms of the number of states and the centers of Gaussian distributions. The levels and periods of different states in inter-state converting traces were identified by hidden Markov modeling (HMM)[16], and the frame data in the same level was pooled to build a histogram and fitted with one or two exponential function(s) in the dwell-time analysis. The transitions between states in the FRET time trajectories were used to build the transition density plot (Fig. 3c).

Nano-positioning system triangulation analysis

Nano-positioning system (NPS) analysis was performed by using the Fast-NPS method[17]. The credible volume accessible by the antenna dye (acceptor) was calculated using the Fast-NPS software, with the parameters of the mean and standard deviation of FRET efficiencies derived from the smFRET histogram, the coordinates of the satellite dye (donor) attached to DNA, the Förster radius of the dye pair, fluorescence anisotropy of both dyes, and the properties of the dye and linker (dye diameter: 13 Å; linker length: 13 Å; linker diameter: 4.5 Å, as suggested in Ref. 17). The coordinates of the satellite dye attachment point were based on the atomic model of Pol III EC (PDB: 5fj8). The Förster radius of the dye pair is 6.0 nm[18,19]. The anisotropy of the donor dye on DNA was measured as ~0.3 using a lifetime technique[11] by means of a time-resolved laser scanning confocal microscope (Q2, ISS, USA) with the measured value in good agreement with those of previous studies[11].

For the anisotropy of the acceptor dye on UAA in Pol III, we adopted the value of ~0.3 reported by the Deniz group[20].

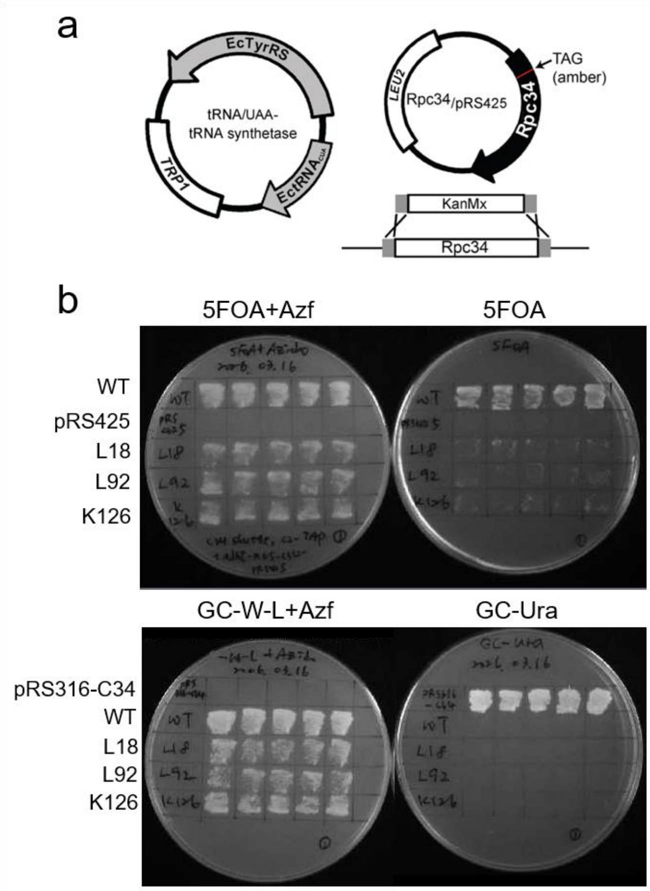

Supplementary Fig. 1 │ Yeast cell plate assays for viability test. (a) The plasmid shuffling strategy used to introduce rpc34 amber replacement mutation in the haploid strain. The Rpc34/pRS425 (Leu2+) plasmid is used to replace the Rpc34/pRS316 (Ura3+) in the 5FOA dropout procedure. The suppressor tRNA/UAA-tRNA synthetase plasmid is used to introduce the UAA into the amber codon in the coding sequence of Rpc34. **(b)** Upper panel, cell growth on 5FOA. With the supplement of AzF, the Rpc34 amber mutants are viable, indicative of successful plasmid shuffling and nonsense suppression. Lower panel, the Rpc34 amber mutants generated from plasmid shuffling (FOA dropout) were not viable on synthetic complete lacking Uracil (GC-Ura), indicative of the complete loss of Ura3+ (Rpc34/pRS316). "GC" stands for synthetic complete with glucose. "W" stands for tryptophan, and "L" leucine. Azf is the abbreviation of azido-L-phenylalanine.

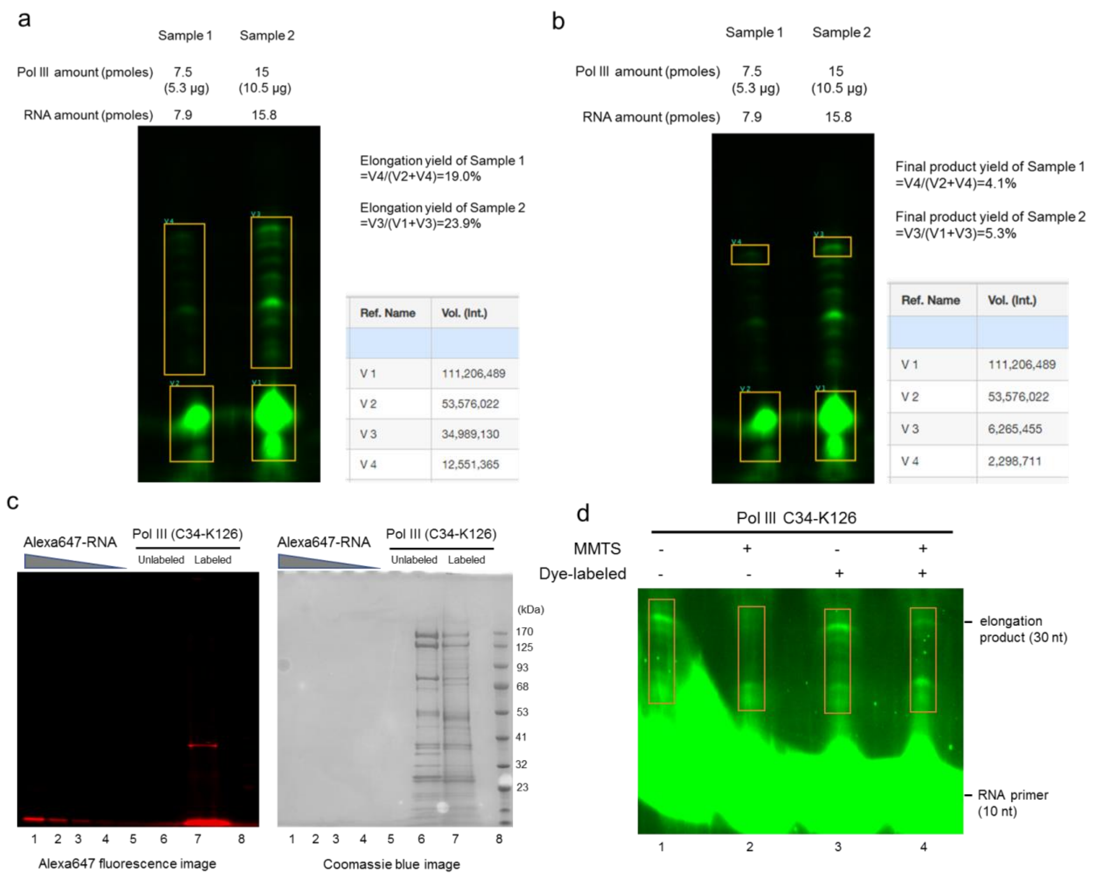

Supplementary Fig. 2 │ Fluorescence-based Pol III elongation assay. (a) Elongation assay of Pol III based on Cy3-RNA primer extension (10 nt). This assay was first established with use of wild-type (WT) Pol III, 7.5 pmol and 15 pmol respectively, where 19.0 % and 23.9% RNA were extended respectively, including all the extended products of different sizes in the orange box. The 30nt product is the full-length product based on the DNA template design. **(b)** is identical to (a). The focused quantitation of the 30nt product shows that it comprises approximately 22% of all products of different sizes. **(c)** DIBO-Alexa647 labeling of mutant Pol III (K126). The left and the right are the same gel scanned by fluorescence and stained by Coomassie Blue: Lane 1, 2, 3,4, are Alexa647 RNA with 2.0, 1.0, 0.5, and 0.2 pmol; Lane 5 is empty, Lane 6 & 7: unlabeled Pol III mutant (K126) (6 pmol), labeled Pol III mutant (K126) "X" pmol. "X" was found to be 3.28 pmol based on the ratio of Lane 7 over Lane 6 (54.8%) in the Coomassie Blue image (right). 6 pmol pol III(K126) initial materials were used to incubate with MMTS followed by DIBO-Alexa647 for selective labeling of Rpc34 K126, but only 3.28 pmol (54.8 %) were recovered). In the fluorescence image (left), the intensities of Lane 1 & 7 are 22,219,473 and 17,033,701. The labeling efficiency is thus calculated to be (17,033,701/22,219,474) * 2/3.28 = 47%. **(d)** Elongation assay for mutant Pol III(K126). In each lane, 12 pmol Pol III(K126) was used. Lane 1, 2, 3, 4 are unlabeled Pol III(K126), MMTS-treated Pol III(K126), DIBO-Alexa647 non-selectively labeled Pol III(K126), and MMTS/DIBO-Alexa647 selectively labeled Pol III(K126). Lane 2, 3, 4 retain 78%, 90% and 84% activity of that of Lane 1 (unlabeled Pol III(K126)).

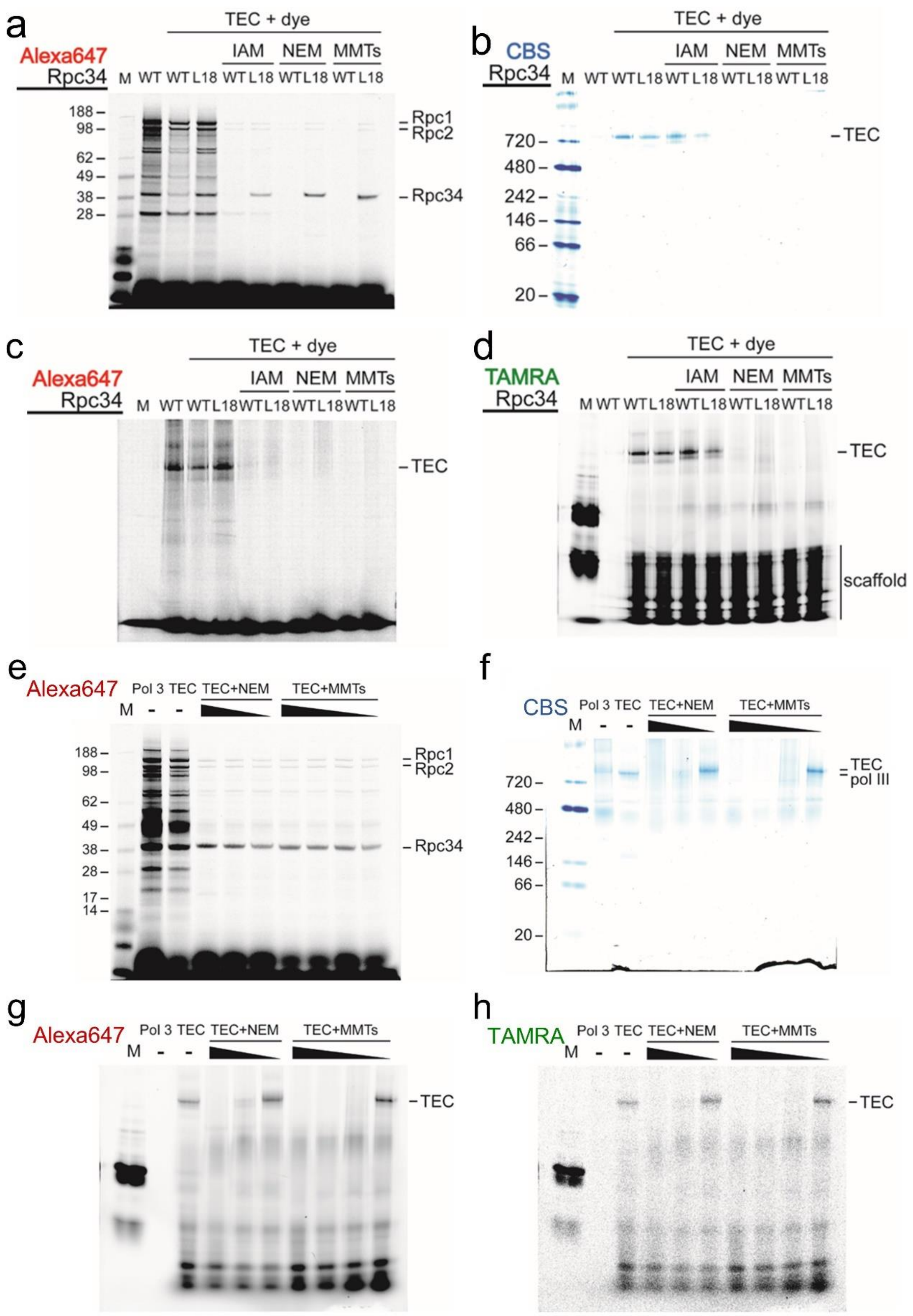
a
Alexa647
Rpc34
TEC + dye
IAM NEM MMTs
M WT WT L18 WT L18 WT L18 WT L18
188
98
62
49
38
28
Rpc1
Rpc2
Rpc34
b
CBS
Rpc34
TEC + dye
IAM NEM MMTs
M WT WT L18 WT L18 WT L18 WT L18
720
480
242
146
66
20
TEC
c
Alexa647
Rpc34
TEC + dye
IAM NEM MMTs
M WT WT L18 WT L18 WT L18 WT L18
TEC
d
TAMRA
Rpc34
TEC + dye
IAM NEM MMTs
M WT WT L18 WT L18 WT L18 WT L18
TEC
scaffold
e
Alexa647
Pol 3 TEC TEC+NEM TEC+MMTs
M - -
188
98
62
49
38
28
17
14
Rpc1
Rpc2
Rpc34
f
CBS
Pol 3 TEC TEC+NEM TEC+MMTs
M - -
720
480
242
146
66
20
TEC
pol III
g
Alexa647
Pol 3 TEC TEC+NEM TEC+MMTs
M - -
TEC
h
TAMRA
Pol 3 TEC TEC+NEM TEC+MMTs
M - -
TEC

Supplementary Fig. 3 │ Selective labeling of incorporated azido-UAA by screening blockers that suppress cross-reactivity between cyclooctynes and thiols in cysteines. (**a**)-(**d**) Thio-reactive molecules (iodoacetamide (IAM), N-ethylmaleimide (NEM) and S-methyl methanethiosulfonate (MMTS)) were tested to suppress non-specific dye labeling. The concentrations of three blockers are 20 mM. Pol III EC was labeled at the azido-UAA in Rpc34(L18) with Alexa647-DIBO and the template DNA was labeled with TAMRA. (**a**) Alexa647 fluorescence image of a SDS-PAGE gel of wild-type and UAA-substituted (Rpc34) Pol III EC. Rpc1 and Rpc2 are the two largest subunits of Pol III and synonymous to Rpc160 and Rpc128, respectively. (**b**) Coomassie blue-stained (CBS) image, (**c**) Alexa647 image and (**d**) TAMRA image of a Native-PAGE gel of wild-type and UAA-substituted (Rpc34) Pol III EC. (**e**)-(**h**) Two blockers were titrated to suppress non-specific dye labeling but permit Pol III EC formation. (**e**) Alexa-647 fluorescence image of a SDS-PAGE gel of Pol III EC with three NEM concentrations (20 mM, 2 mM and 0.2 mM) and four MMTS concentrations (20 mM, 2 mM, 0.2 mM and 20 μM). Note that NEM or MMTS at the lowest concentration (0.2 mM for NEM and 20 μM for MMTS) proved sufficient to reduce non-specific dye labeling. (**f**) CBS image, (**g**) Alexa647 image and (**h**) TAMRA image of a Native-PAGE gel of Pol III EC with three NEM concentrations (20 mM, 2 mM and 0.2 mM) and four MMTS concentrations (20 mM, 2 mM, 0.2 mM and 20 μM). Note that 0.2 mM NEM or 20 μM MMTS allowed formation of Pol III EC.

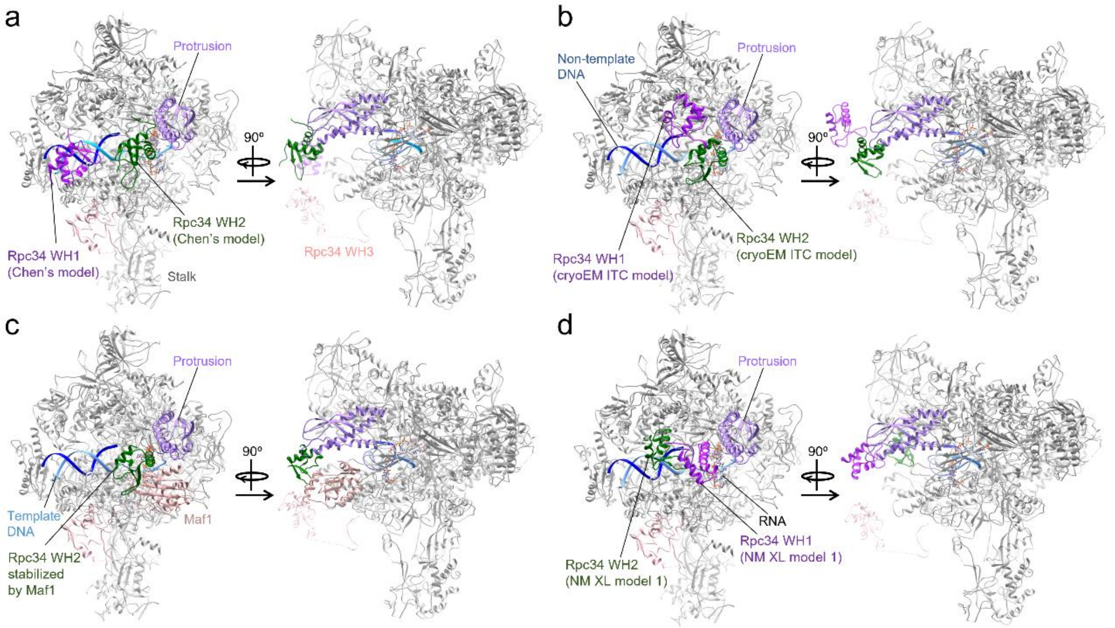

e

Structural model	Distances between the mean dye positions R_{MP} (Donor: T-DNA(+7), Acceptor: Rpc34-K126) (Å)	Predicted FRET efficiency
PA-XL	72.5	0.21
Cryo-EM ITC	70.4	0.24
Pol III with Maf1	68.9	0.26
NM XL model 1	46.2	0.80

Supplementary Fig. 4 │ Positions of Rpc34 tandem-winged-helix (tWH) domain in various models of Pol III in different contexts from literature. (**a**) Rpc34 WH2 (dark green) in Chen's photo-activated crosslinking (PA-XL) model[21], (**b**) Rpc34 WH2 in the initial transcribing complex (ITC, PDB: 6f41)[22,23], (**c**) Rpc34 WH2 stabilized by Maf1 (PDB: 6tut)[24], and (**d**) Rpc34 WH2 in Nilges & Muller crosslinking (NM-XL) Model 1[25]. All models were docked into Pol III elongation complex model (gray, PDB: 5fj8) based on alignment of Rpc1 between models. (**e**) The distances between the mean dye positions R_{MP} and the predicted FRET efficiency in various Pol III models. The donor dye and acceptor are labeled at T-DNA(+7) and Rpc34-K126, respectively.

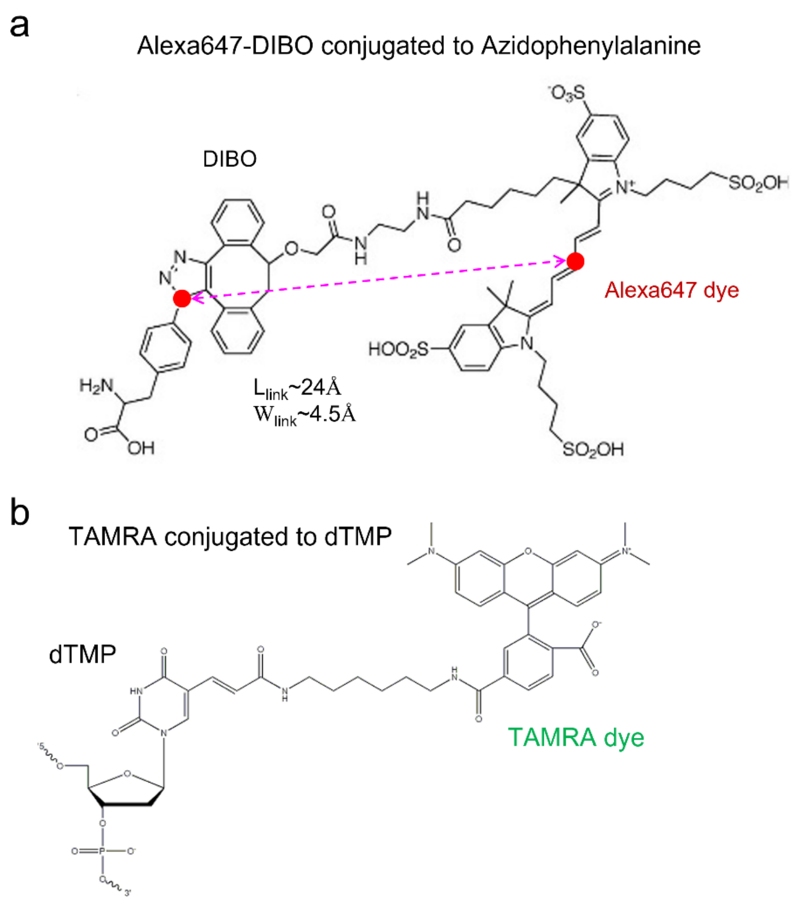

Supplementary Fig. 5 │Chemical structure of dye linker for DIBO-Alexa647 and TAMRA used in the present study. (**a**) DIBO-Alexa647. (b) TAMRA dye linked to the base of a Thymine (T).

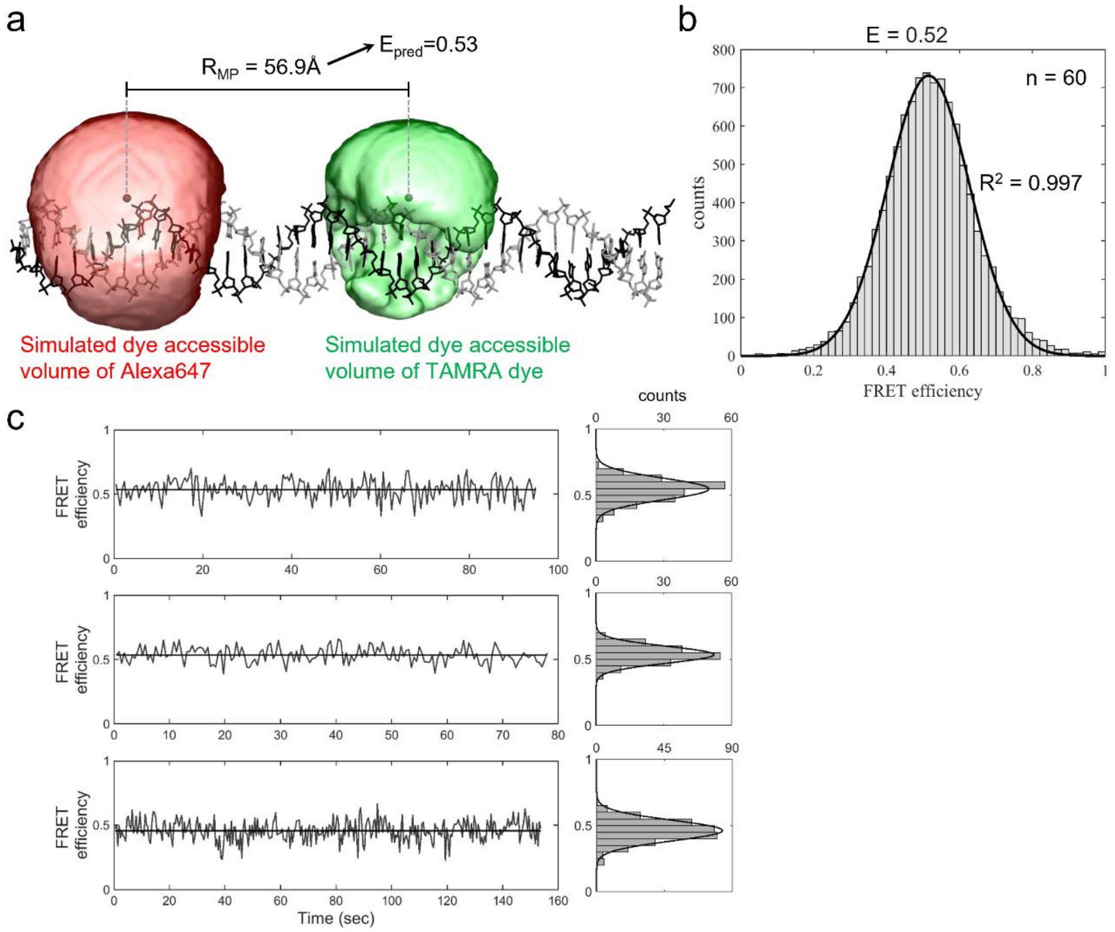

Supplementary Fig. 6 │SmFRET measurement of double-labeled benchmark DNA against predicted FRET efficiency based on modeling with dye-swirling. (**a**) Dye accessible volumes of Alexa647 and TAMRA were simulated by FPS software[10]. The DNA sequence and the dye labeling positions of the benchmark DNA was the same as those used in Ref. 11, where the two nucleotides labeled by dye were separated by 15 bp. The DNA model was built by using Web 3DNA[12]. The predicted FRET efficiency E_{pred} of 0.53 was calculated from the distance between the centers of two dye accessible volumes R_{MP}. (**b**) The measured smFRET efficiency histogram of double-labeled benchmark DNA fitted with single Gaussian function shows the center of distribution (E=0.52), agreeing well with the predicted FRET efficiency (E=0.53). (**c**) *Left*: Three representative time trajectories of double-labeled benchmark DNA show stationary FRET level as expected. *Right*: The FRET efficiency histograms of corresponding to the trajectories in the left panel. The distribution in each FRET efficiency histogram was best fitted with single Gaussian distribution.

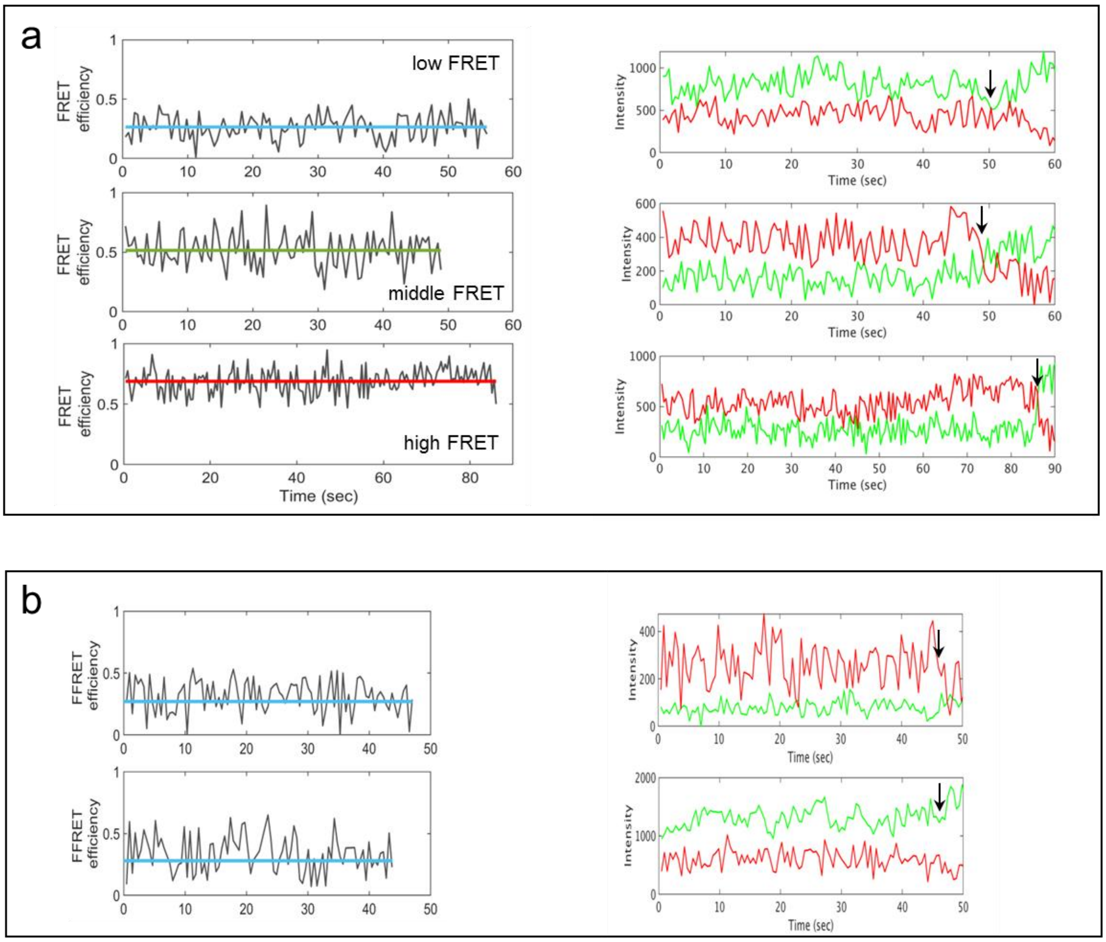

Supplementary Fig. 7 │FRET traces with the donor/acceptor traces. (a) this panel contain those three corresponding to those three in **Fig. 2b**. **(b)** this panel contains those two corresponding to those two in **Fig. 2d**. Black arrows denote where acceptor photo-bleaching events occur that terminate FRET traces.

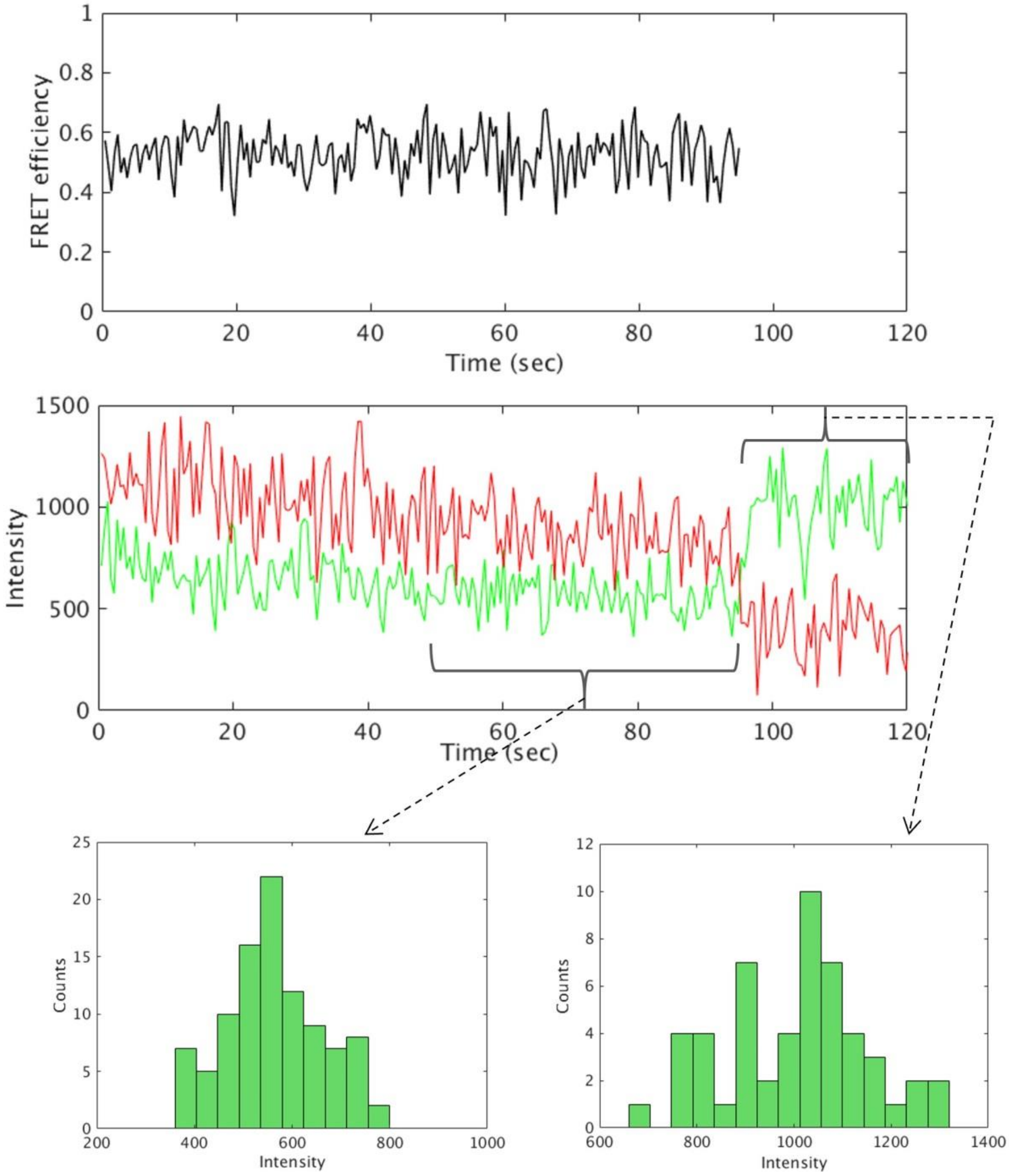

Supplementary Fig. 8 │Photon statistics analysis of shot-noise limited smFRET time traces from a single-molecule of benchmark DNA corresponding to (**a**) in **Supplementary Fig. 5**, including its FRET, donor and acceptor traces before and after photo-bleaching. The intensity histograms of the donor before and after acceptor photo-bleached show the intensity variations roughly scale with square root of the mean intensities, consistent with the shot-noise limited photon statistics.

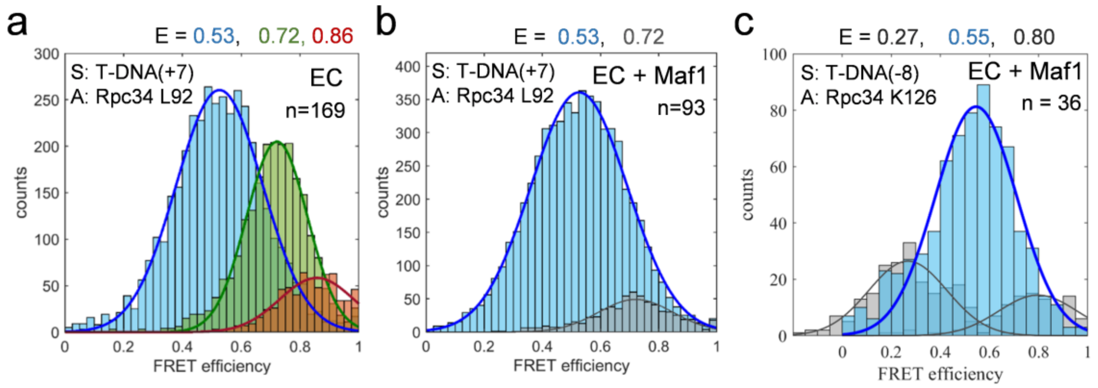

d

Satellite: T-DNA(-8)
Antenna: Rpc34 K126

Satellite: T-DNA(+7) / Antenna: Rpc34 K126

	E=0.23	E=0.50	E=0.78
E=0.27	×	×	Distal
E=0.52	×	Middle	×
E=0.68	Proximal	×	×

Satellite: T-DNA(-8)
Antenna: Rpc34 L92

Satellite: T-DNA(+7) / Antenna: Rpc34 L92

	E=0.25	E=0.39	E=0.59
E=0.53	×	×	Distal
E=0.72	×	Middle	×
E=0.86	Proximal	×	×

Supplementary Fig. 9 │ FRET efficiency histograms of Rpc34 WH2 in Pol III EC without or with Maf1. (**a**) The FRET efficiency histogram of Rpc34 WH2 in Pol III EC probed by a donor-acceptor (D/A) pair at DNA(+7)/Rpc34 L92 (+7/92) (Same as the histogram in the bottom left in Fig. 4b). (**b**) The FRET efficiency histogram of Rpc34 WH2 in Pol III EC with Maf1 probed by D/A pair +7/92. The major population centered at E=0.53 indicates the FRET state of Maf1-stabilized Rpc34 WH2. E: the center of FRET efficiency distribution; D: donor; A: acceptor; n: number of molecules. (**c**) The FRET efficiency histogram of Rpc34 WH2 in Pol III EC with Maf1 probed by D/A pair -8/126. The FRET efficiency (E=0.55) of the major population matches well with the predicted FRET efficiency (E=0.54 as shown in Supplementary Table 3). (**d**) The matrices of satellite-antenna pairs for NPS analysis where the antenna dye was labeled at K126 (left panel) or L92 (right panel).

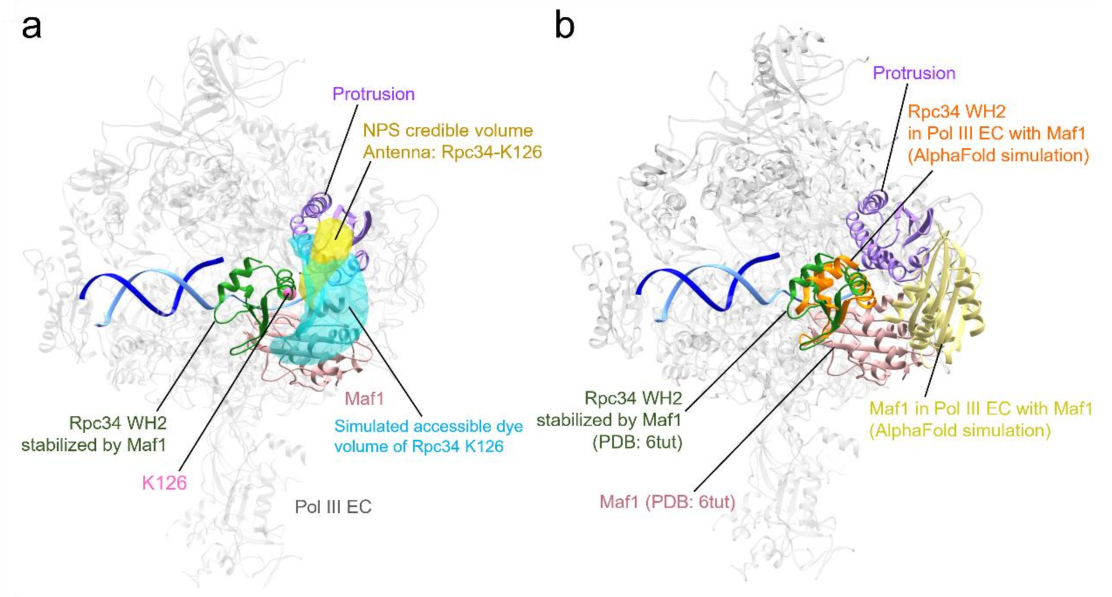

Supplementary Fig. 10 │ Experimental NPS credible volume and simulated accessible dye volume for Maf1-stabilizaing Rpc34 WH2. (a) The position of Rpc34 WH2 is situated by stabilization via Maf1 (PDB: 6tut)[24]. The antenna dye was labeled at Rpc34 K126 (hot pink sphere). The experimental NPS credible volume (yellow) was obtained by using two independent smFRET measurements from two S/A pairs: +7/126 and -8/126 in Pol III EC with addition of Maf1 (Fig. 2c and Extended Data Fig. 3c). In the absence of a solved Pol III-EC-Maf1 cryo-EM structure, we simulated the accessible dye volume (cyan) of antenna dye attached to Rpc34 K126 based on the Pol III EC model (PDB: 5fj8) docked with Rpc34 WH2 and Maf1 positions in Pol III-Maf1 structure (PDB: 6tut) [24]. The simulation was performed by using FPS software[10] and known dye parameters (size of dye and the length and width of linker) (Supplementary Table 2). **(b)** During the manuscript preparation, AlphaFold emerged. We thereby used it to predict a structure for Pol III-EC-Maf1 complex. This predicted structure shows the position of Rpc34 WH2 does not change significantly compared to that in Pol III-Maf1 (PDB: 6tut) [24]. However, Maf1 seems to be re-arranged by the DNA scaffold. Importantly, that the positions of Rpc34 WH2 are highly similar in the two models, one from cryo-EM experiment and the other from AlphaFold prediction, warrants the plausibility of our accessible dye modeling based on the Pol III-Maf1 structure (PDB: 6tut) [24].

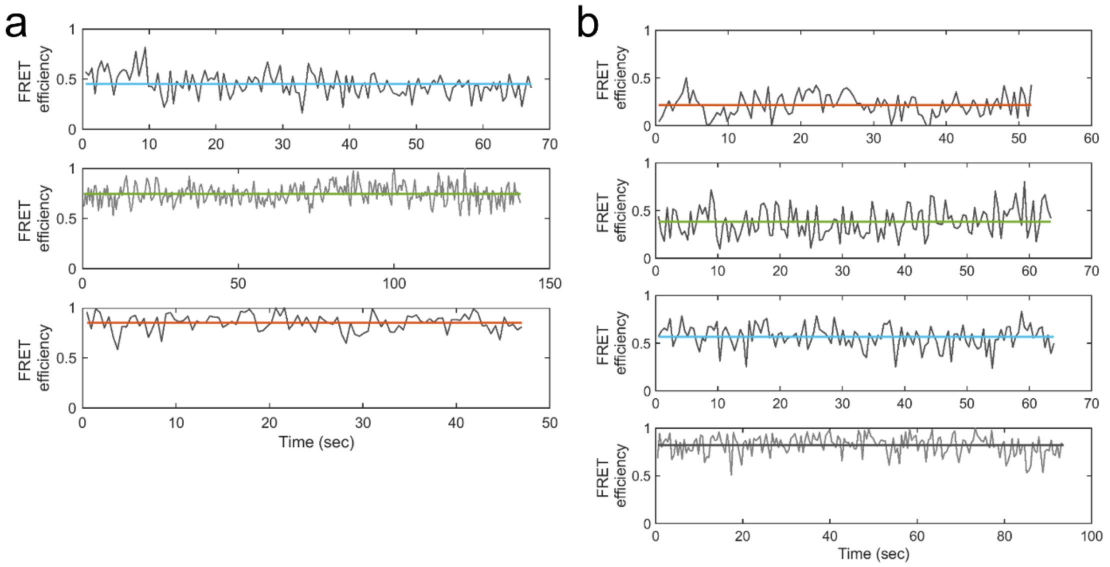

Supplementary Fig. 11 │ The raw representative time trajectories of stationary FRET states of Rpc34 WH2 in Pol III EC, where the acceptor dye was labeled at Rpc34 L92. (**a**) The three trajectories correspond to the populations of E=0.53, E=0.72 and E=0.86 in the FRET efficiency histogram of Rpc34 WH2 in Pol III EC probed by donor-acceptor (D/A) pair +7/92 (bottom left in Fig. 4b). The three lines (blue, green and orange) represent the average FRET efficiencies of each time trajectory. (**b**) The four trajectories correspond to the populations of E=0.25, E=0.39, E=0.59 and E=0.81 in the FRET efficiency histogram of Rpc34 WH2 in Pol III EC probed by D/A pair -8/92 (bottom right in Fig. 4b). The four lines (orange, green, blue and gray) represent the mean FRET efficiencies of each time trajectory.

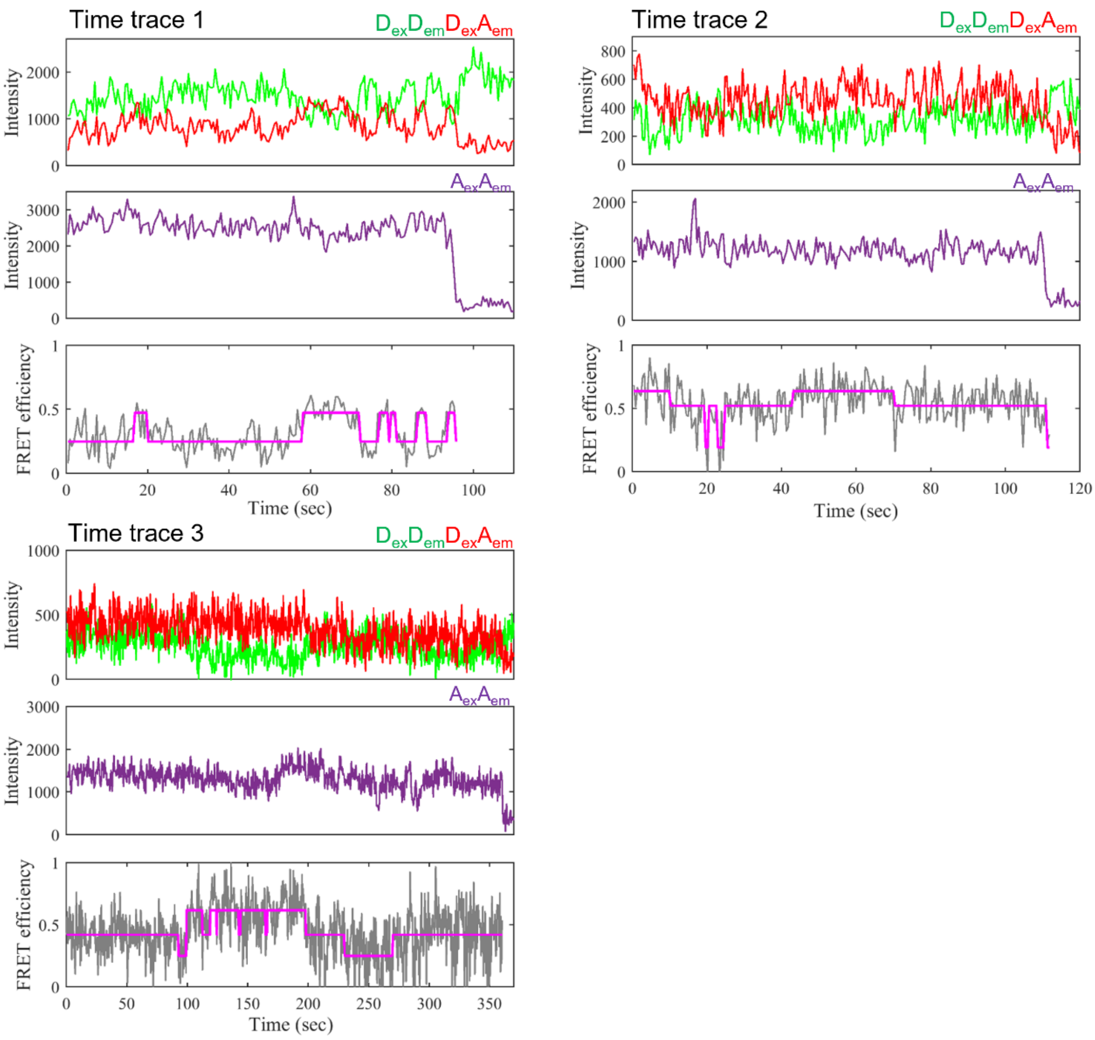

Supplementary Fig. 12 │ Three representative time trajectories of inter-state switching FRET with ALEX channel (Time traces 1-3 in Fig. 3a). For each single-molecule time trace, three panels corresponding to three channels are shown. The upper panel represents the donor intensity (D_{em}), colored in green, when donor is excited using 532 nm laser light (D_{ex}), and the acceptor intensity (A_{em}), colored in red, also when donor is excited. The middle panel represents the acceptor intensity (A_{em}), colored in purple, when acceptor is excited using 638 nm laser light (A_{ex}). The lower panel represents the corresponding time traces of FRET efficiencies (gray trace) that are calculated from D_{em} and A_{em} from the upper panel. Hidden Markov modeling (HMM) is used for identifying the level of each FRET state, colored in magenta, and the respective durations.

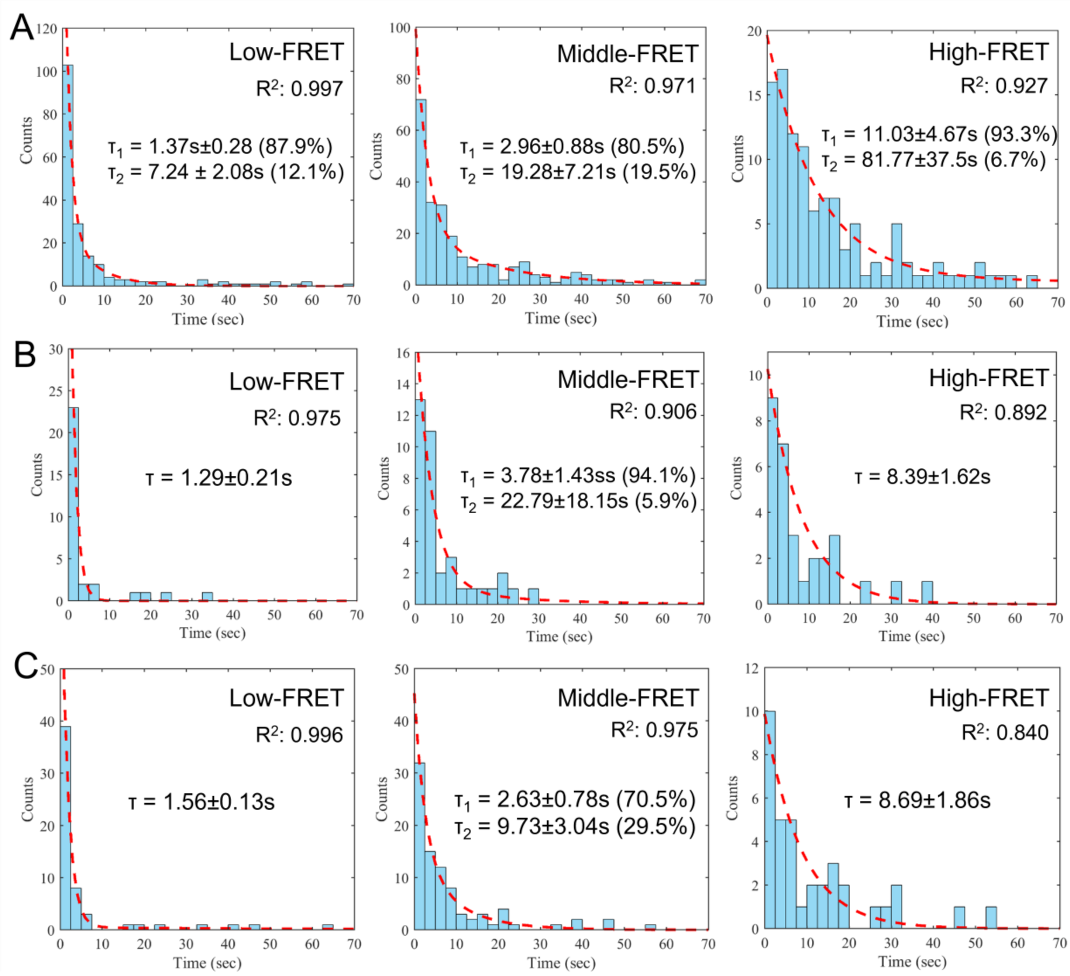

Supplementary Fig. 13 │ The dwell time histograms for low-, middle-, and high-FRET efficiencies in the three individual smFRET measurements. TAMRA was labeled at the +7 position of the template DNA. DIBO-Alexa647 was conjugated to Rpc34 K126 via the strain-promoted azide-alkyne [3+2] cycloaddition (SpAAC), namely copper-free click chemistry. **(a)** The dwell time histogram was built from time durations extracted by HHM analysis from 62 inter-state switching time traces out of 110 time-traces in the first experiment. **(b)** The dwell time histogram was built from time durations extracted by HHM analysis from 25 inter-state switching time traces out of 80 time-traces in the second experiment. **(c)** The dwell time histogram was built from time durations extracted by HHM analysis from 18 inter-state switching time traces out of 54 time-traces in the third experiment. The dwell time histograms were well fitted with one or two exponential function(s). In the latter case, the exponential function with shorter life time (τ) always dominates. The average dwell times for low-, middle- and high-FRET are 1.41±0.11 sec, 3.12±0.48 sec and 9.37±1.18 sec, respectively.

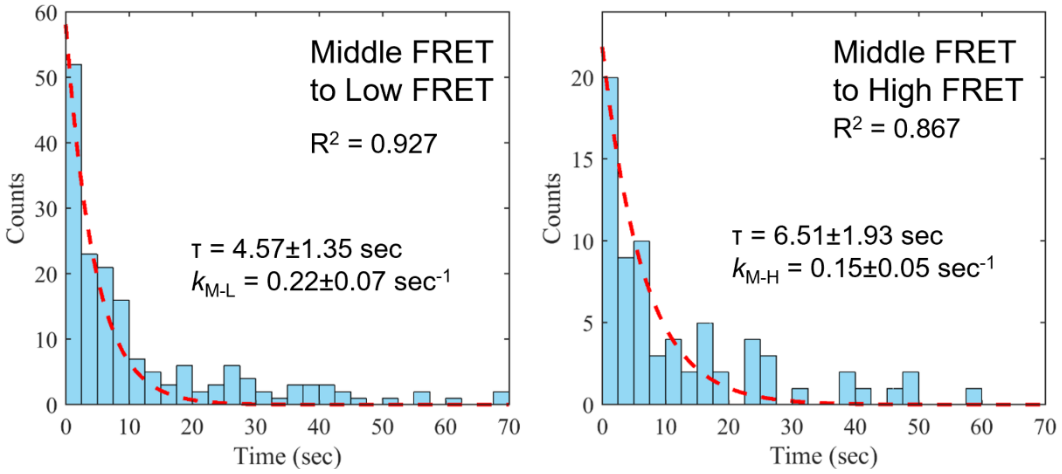

Supplementary Fig. 14 │ The dwell time histograms for the middle to low FRET transition and the middle to high FRET transition. The dwell time data for the middle FRET were separated into two groups based on the transition type: from the middle to low FRET, or from the middle to high FRET. Both histograms were best fitted to single exponential function. τ: dwell time; $k_{M\text{-}L}$: transition rate from middle to low FRET; $k_{M\text{-}H}$: transition rate from middle to high FRET.

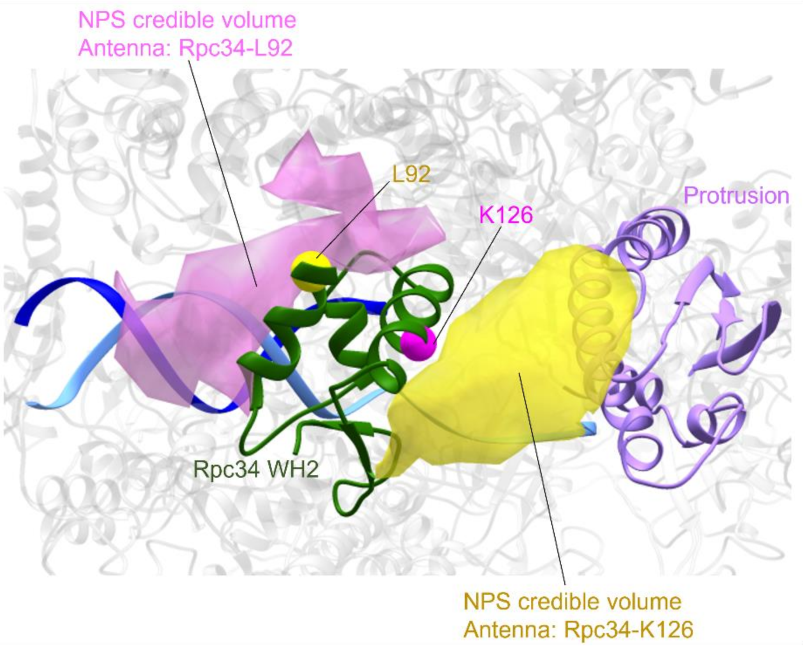

Supplementary Fig. 15 │ Docking of the Rpc34 WH2 domain based on two NPS credible volumes for K126 and L92. We manually positioned and orientated the Rpc34 WH2 domain between the two NPS credible volumes by (1) positioning the Rpc34 WH2 domain to let the ball (in pink) representing dye attachment point at K126 contact the middle region of the corresponding dye NPS volume surface (in yellow), (2) rotating the domain to let the ball (in yellow) representing dye attachment point at L92 contact the middle region of the corresponding dye NPS volume surface (in pink), (3) fine-tuning the position and orientation of the domain to simultaneously minimizing the distance between the pink ball and the center of yellow volume, and that between the yellow ball and the center of pink volume. To illustrate this docking, we employed the middle site docked by Rpc34 WH2. The WH2 domain was localized and flanked by two NPS credible volumes: one from Rpc34 L92, obtained from the S/A pairs (+7/92, -8/92), and the other from K126, obtained from (+7/126, -8/126). The position and orientation of WH2 were thus optimized by this docking procedure.

Supplementary Table 1 │ Number of total and exposed cysteines on the Pol III surface

Subunit	Number of total cysteines	Number of exposed cysteines* (PDB: 5fj8)	Number of exposed cysteines within FRET sensing range
Rpc1(160)	24	6	5
Rpc2(128)	19	4	3
Rpc82	4	2	2
Rpc53	0	0	0
AC40	5	3	0
Rpc37	0	0	0
Rpc34	3	0	0
Rpc31	0	0	0
ABC27	3	2	2
Rpc25	4	2	0
Rpc17	1	0	0
ABC14.5	2	1	0

*The exposed cysteines were determined by the property of relative amino acid exposure in UCSF Chimera (Ref: https://www.cgl.ucsf.edu/chimera/docs/UsersGuide/surfnorm.html) with a threshold of 0.6 (where the value of this property ranged from 0 to 1.2).

Supplementary Table 2 │ Predicted FRET efficiencies of FRET pair +7/126 and -8/126 in different structural models of Pol III.

Structural model	Distances between the mean dye positions R_{MP} of pair +7/126* (Å)	Predicted FRET efficiency of pair +7/126†	Distances between the mean dye positions R_{MP} of pair -8/126* (Å)	Predicted FRET efficiency of pair -8/126†
Pol III with Maf1[25]	68.9	0.26	56.4	0.54
Cryo-EM ITC[23]	70.4	0.24	48.1	0.76
Cryo-EM PIC (Muller)[23]	69.6	0.25	47.9	0.76
Cryo-EM PIC (Vannini)[24]	67.1	0.30	45.5	0.81
PA-XL[22]	72.5	0.21	59.0	0.48
NM XL model 1[26]	46.2	0.80	57.9	0.50
NM XL model 2[26]	51.6	0.67	49.5	0.72
NM XL model 3[26]	46.5	0.79	43.7	0.85
NM XL model 4[26]	40.0	0.90	60.1	0.45

*The dye attached positions of donor and acceptor of FRET pair +7/126 are the atom C7 of +7 position in template DNA and the atom CD of Rpc34-K126, respectively. The dye attached positions of donor and acceptor of FRET pair -8/126 are the atom C5 of -8 position in template DNA and the atom CD of Rpc34-K126, respectively.
†The estimation of FRET efficiency is based on the Forster radius 58.1Å of the dye pair TAMRA and Alexa647.

Supplementary Table 3 │ The dye parameters for the simulation of dye accessible volumes.

Dye reagent for labeling	Length of linker, L_{link} (Å)	Width of linker, W_{link} (Å)	Dimension 1 of dye, $R_{dye(1)}$ (Å)	Dimension 2 of dye, $R_{dye(2)}$ (Å)	Dimension 3 of dye, $R_{dye(3)}$ (Å)
Alexa647-DIBO	24.0	4.5	11.0	4.7	1.5
TAMRA	20.0	4.5	5.0	4.0	1.5
DyLight650-phosphine	22.5	4.5	11.0	4.7	1.5

*Dye parameters were suggested from the manual of FPS software and estimated from chemical structure of dye reagents.

Supplementary Table 4 │ Predicted FRET efficiencies of FRET pair +7/92 and -8/92 in different structural models of Pol III.

Structural model	Distances between the mean dye positions R_{MP} of pair +7/92* (Å)	Predicted FRET efficiency of pair +7/92†	Distances between the mean dye positions R_{MP} of pair -8/92* (Å)	Predicted FRET efficiency of pair -8/92†
Pol III with Maf1[25]	51.7	0.63	61.8	0.37
Cryo-EM ITC[23]	56.4	0.50	54.6	0.55
Cryo-EM PIC (Muller)[23]	59.9	0.42	57.9	0.47
Cryo-EM PIC (Vannini)[24]	60.9	0.39	60.4	0.40
PA-XL[22]	43.2	0.83	48.3	0.72
NM XL model 1[26]	32.2	0.97	71.2	0.20
NM XL model 2[26]	55.2	0.54	65.8	0.29
NM XL model 3[26]	64.4	0.32	69.6	0.22
NM XL model 4[26]	54.4	0.56	47.5	0.74

*The dye attached positions of donor and acceptor of FRET pair 3 are the atom C7 of +7 position in template DNA and the atom CG of Rpc34-L92, respectively. The dye attached positions of donor and acceptor of FRET pair 4 are the atom C5 of -8 position in template DNA and the atom CG of Rpc34-L92, respectively.
†The estimation of FRET efficiency is based on the Forster radius 56.6Å of the dye pair TAMRA and DyLight650.

References

1. Chin, J. W., et al. An expanded eukaryotic genetic code. *Science* **301**, 964-967 (2003).
2. Chen, S., Schultz, P. G. & Brock, A. An improved system for the generation and analysis of mutant proteins containing unnatural amino acids in Saccharomyces cerevisiae. J. Mol. Biol. 371, 112-122 (2007).
3. Wei, Y. Y. & Chen, H. T. Functions of the TFIIE-related tandem winged-helix domain of Rpc34 in RNA polymerase III initiation and elongation. *Mol. Cell. Biol.* **38**, e00105-17 (2018).
4. Wu, J. S., et al. Deriving a sub-nanomolar affinity peptide from TAP to enable smFRET analysis of RNA polymerase II complexes. *Methods* **159-160**, 59-69 (2019).
5. Roy, R., Hohng, S. & Ha, T. A practical guide to single-molecule FRET. *Nat. Methods* **5**, 507-516

(2008).

6. Dave, R., Terry, D. S., Munro, J. B. & Blanchard, S. C. Mitigating unwanted photophysical processes for improved single-molecule fluorescence imaging. *Biophys. J.*, **96**, 2371-2381 (2009).
7. Rasnik, I., McKinney, S. A. & Ha, T. Nonblinking and long-lasting single-molecule fluorescence imaging. *Nat. Methods*, **3**, 891-893 (2006).
8. Kapanidis, A. N., et al. Fluorescence-aided molecule sorting: Analysis of structure and interactions by alternating-laser excitation of single molecules. *Proc. Natl. Acad. Sci. USA* **101**, 8936-8941 (2004).
9. Lee, S., Lee, J. & Hohng, S. Single-molecule three-color FRET with both negligible spectral overlap and long observation time. *PLoS One* **5**, e12270 (2010).
10. Kalinin, S., et al. A toolkit and benchmark study for FRET-restrained high-precision structural modeling. *Nat. Methods* **9**, 1218-1225 (2012).
11. Hellenkamp B., et al., Precision and accuracy of single-molecule FRET measurements—a multi-laboratory benchmark study, *Nat. Methods* **15**, 669-676 (2018).
12. Li, S. and Lu, X. J., Web 3DNA 2.0 for the analysis, visualization, and modeling of 3D nucleic acid structures, *Nucleic Acids Res.* **47**, W26-W34 (2019).
13. Lee, H. C., Lin, B. L., Chang, W. H. & Tu, I. P. Toward automated denoising of single molecular Förster resonance energy transfer data. *J. Biomed. Opt.* **17**, 011007 (2012).
14. McCann, J. J., Choi, U. B., Zheng, L., Weninger, K. & Bowen, M. E. Optimizing methods to recover absolute FRET efficiency from immobilized single molecules. *Biophys. J.* **99**, 961-970 (2010).
15. Taylor, J. N., Makarov, D. E. & Landes, C. F. Denoising single-molecule FRET trajectories with wavelets and Bayesian inference. *Biophys J.* **98**, 164-73 (2010).
16. McKinney, S. A., Joo, C. & Ha, T. Analysis of single-molecule FRET trajectories using hidden Markov modeling. *Biophys J.* **91**, 1941-1951 (2006).
17. Nagy, J., Eilert, T. & Michaelis, J. Precision and accuracy in smFRET based structural studies — A

benchmark study of the Fast-Nano-Positioning System. *J. Chem. Phys.* **148**, 123308 (2018).

18. Sass, L. E., Lanyi, C., Weninger, K. & Erie, D. A. Single-molecule FRET TACKLE reveals highly dynamic mismatched DNA-MutS complexes. *Biochemistry* **49**, 3174–3190 (2010).
19. Treutlein, B., Dynamic architecture of a minimal RNA polymerase II open promoter complex. *Mol. Cell* **46**, 136-146 (2012).
20. Kim, J., et al. Simple and efficient strategy for site-specific dual labeling of proteins for single-molecule fluorescence resonance energy transfer analysis. *Anal. Chem.* **85**, 1468–1474 (2013).
21. Wu, C. C., et al. RNA polymerase III subunit architecture and implications for open promoter complex formation. *Proc. Natl. Acad. Sci. USA* **109**, 19232–19237 (2012).
22. Vorländer, M. K., Khatter, H., Wetzel, R., Hagen, W. J. H. & Müller, C. W. Molecular mechanism of promoter opening by RNA polymerase III. *Nature* **553**, 295-300 (2018).
23. Abascal-Palacios, G., Ramsay, E. P., Beuron, F., Morris, E. & Vannini, A. Structural basis of RNA polymerase III transcription initiation. *Nature* **553**, 301-306 (2018).
24. Vorländer, M. K., et al. Structural basis for RNA polymerase III transcription repression by Maf1. *Nat Struct Mol Biol.*, **27**, 229-232 (2020).
25. Ferber, M., et al. Automated structure modeling of large protein assemblies using crosslinks as distance restraints. *Nat. Methods*, **13**, 515-520 (2016).
